# Locus coeruleus α-synuclein overexpression induces prodromal Parkinsonian features in mice

**DOI:** 10.1038/s41531-026-01420-w

**Published:** 2026-06-08

**Authors:** Jone Razquin, Laura De Las Heras-García, Andrea Vaquero-Rodríguez, Sara Fernández-Roqueñí, Gloria González-Aseguinolaza, Edgar Soria-Gómez, Jérôme Baufreton, Harkaitz Bengoetxea, Leticia Abecia, Jorge E. Ortega, Naiara Ortuzar, Cristina Miguélez

**Affiliations:** 1 Department of Pharmacology, Faculty of Medicine and Nursing, University of the Basque Country UPV/EHU, Leioa, Spain; 2 Department of Neuroscience, Faculty of Medicine and Nursing, University of the Basque Country UPV/EHU, Leioa, Spain; 3 Neurodegenerative Diseases Group, Biobizkaia Health Research Institute, Barakaldo, Spain; 4 Univ. Bordeaux, CNRS, IMN, UMR 5293, Bordeaux, France; 5 Gene Therapy for Rare Diseases Program, DNA & RNA Medicine Division, CIMA, Universidad de Navarra, Pamplona, Navarra, Spain; 6 Achucarro Basque Center for Neuroscience, Science Park of the UPV/EHU, Leioa, Spain; 7 IKERBASQUE, Basque Foundation for Science, Bilbao, Spain; 8 Department of Immunology, Microbiology and Parasitology, University of the Basque Country UPV/EHU, Leioa, Spain; 9 Centro de Investigación Biomédica en Red de Salud Mental, Instituto de Salud Carlos III, Leioa, Spain; 10 Early Phases of Psychosis Group, Biobizkaia Health Research Institute, Barakaldo, Spain; 11 Present Address: Department of Neuroscience, Karolinska Institutet, Stockholm, Sweden

**Keywords:** Neurology, Neuroscience

## Abstract

The locus coeruleus is among the earliest brain regions affected by α-synuclein pathology in prodromal Parkinson’s disease, contributing to non-motor symptoms, yet its early pathogenic role remains unclear. We developed a mouse model in which human α-synuclein was selectively overexpressed in noradrenergic locus coeruleus neurons using PRSx8-driven adeno-associated viral vector. Mice were analysed at 1 and 3 months post-injection using histological, behavioural, neurochemical and electrophysiological approaches. Monomeric α-synuclein was localized in the locus coeruleus cell bodies and distributed throughout noradrenergic neuronal projections, while phosphorylated α-synuclein was predominantly confined to the locus coeruleus somata. We also observed reduced noradrenergic axonal density and early decreases in noradrenaline and serotonin levels without neuron loss. In contrast, the dopaminergic system remained unchanged. Electrophysiological recordings revealed transient, sex-dependent alterations in neuronal excitability. At the behavioural level, mice exhibited cognitive deficits and altered innate-emotional behaviour persisting over time. Sex-specific analyses revealed shortening of the colon in females, in the absence of detectable morphological alterations, α-synuclein deposition, or systemic inflammation. This model recapitulates some of the key non-motor Parkinson’s disease features, highlighting the locus coeruleus as a potential target for early intervention.

**Figure:**
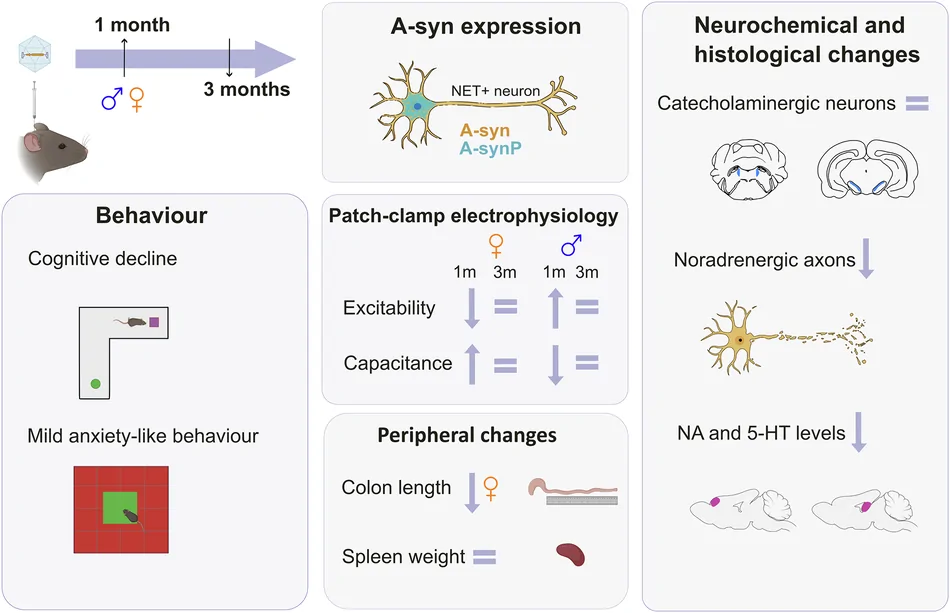

## Introduction

Parkinson’s disease (PD) is the most prevalent movement disorder and the second most common neurodegenerative disease worldwide, affecting approximately 0.1% of the global population^1^. Its pathological hallmark is the accumulation of α-synuclein (α-syn) aggregates, forming Lewy bodies, which compromise motor nuclei such as the substantia nigra pars compacta (SNc), resulting in the characteristic motor symptoms of bradykinesia, tremor, and rigidity. However, Lewy body pathology also develops in non-motor regions up to two decades before motor symptoms emerge, particularly in the locus coeruleus (LC), one of the earliest brainstem structures to be affected^2,3^.

Although LC degeneration is more variable than that of the SNc^4,5^, post-mortem studies consistently show significant LC neuronal loss and α-syn pathology, with LC involvement potentially preceding dopaminergic degeneration in the SNc in some PD phenotypes^2,6,7^. Moreover, neuromelanin-sensitive MRI studies reveal progressive loss of LC signal in both genetic and idiopathic PD^8^. Noradrenergic dysfunction affects a broad range of projection targets, including the prefrontal cortex (PFC), motor cortices, striatum, thalamus, hypothalamus, and cerebellum^9^, as well as the peripheral autonomic system^10^. This disruption is reflected in reduced cerebrospinal fluid levels of the noradrenaline (NA) metabolite dihydroxyphenylglycol and decreased dopamine-β-hydroxylase (DBH) activity^11^.

Noradrenergic impairment has been linked to several non-motor symptoms of PD, including autonomic dysfunction, depression, anxiety, and cognitive decline^12^. Orthostatic hypotension, urinary, and sexual dysfunction are correlated with reduced LC integrity and lower NA levels^9^. Similarly, LC degeneration is strongly associated with neuropsychiatric disturbances. Depressed PD patients exhibit greater noradrenergic deficits than non-depressed patients. This has been observed through lower contrast-to-noise ratios, neuronal loss and gliosis in the LC^13,14^ along with reduced NA and DA innervation of limbic areas^15^. Imaging studies have also demonstrated that LC degeneration is associated with higher apathy scores^16^ and cognitive impairment^17^, particularly affecting attention and working memory domains^18^.

Experimental studies also highlight the LC as a critical modulator of disease progression. In PD models, NA depletion exacerbates nigrostriatal degeneration^19^ whereas enhancing noradrenergic tone promotes SNc neuronal survival^20^. Preclinical approaches using combined NA/DA lesions or genetic models provide additional evidence for a key role of LC dysfunction in the emergence of non-motor PD features^21,22^. These findings closely mirror the deficits observed in patients.

Together, clinical and preclinical data underscore a crucial role for noradrenergic dysfunction in PD pathogenesis. However, the contribution of α-syn accumulation specifically within the LC to the early features of the disease remains unclear. Here, we investigate the impact of adeno-associated virus (AAV)-mediated selective human α-syn overexpression in LC neurons, assessing its contribution to noradrenergic dysfunction and the emergence of prodromal PD symptoms.

## Results

### Targeted α-syn overexpression in LC neurons and propagation along noradrenergic projections

**Fig. 1:**
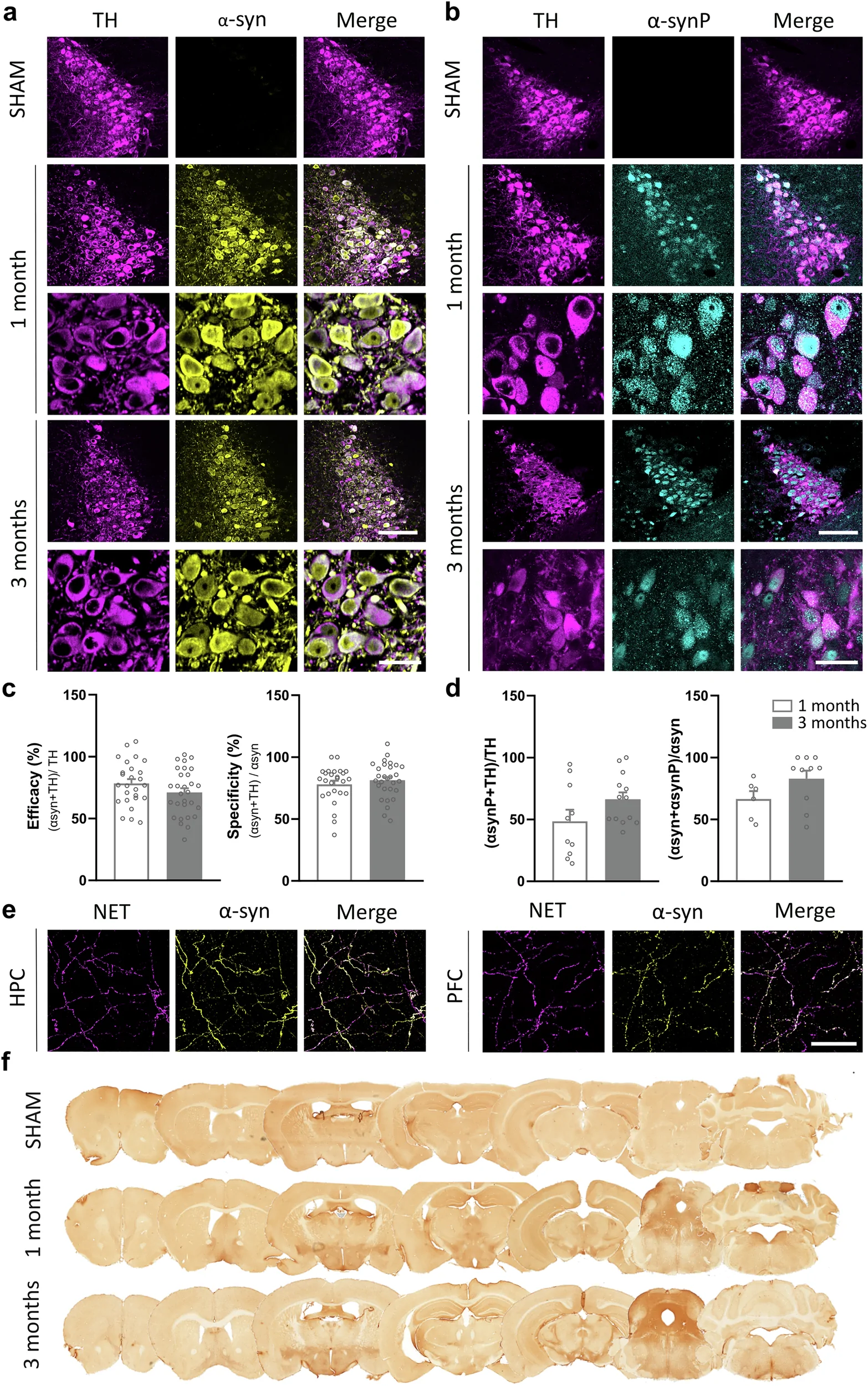
Α-syn expression in the locus coeruleus. **a** Representative imagesshowing colocalizationof TH+ (magenta) and α-syn (yellow) in the LC at 1 and 3 months post-surgery. **b** Phosphorylated α-syn (cyan) remains restricted to LC neuronal somata. Scale bars: 100 μm (×20), 25 μm (×63). **c** Efficacy at 1 and 3 months post-injection, defined as the ratio of neurons positive for both α-syn and TH to the total number of TH⁺ neurons (α-syn+TH)/(TH) and specificity, defined asthe ratio of neurons positive for both α-syn and TH to the total number of α-syn neurons(α-syn+TH)/ (α-syn).**d**Colocalization analysis of α-synP neurons in the locus coeruleus. **e** Colocalization of α-syn withnorepinephrine transporter (NET) in the hippocampus and prefrontal cortex. Scale bars: 25 μm (×63). **f** Distribution of α-syn along the rostro-caudal axis in bilaterally injected brains.

To evaluate the efficiency of AAV-PRSx8-hsyn mediated α-syn overexpression, immunofluorescence analyses were performed in brain tissue samples collected 1 and 3 months after injection. Α-syn was selectively expressed in tyrosine hydroxylase (TH) + neurons of the LC at both time points. High-magnification images confirmed its localization within noradrenergic neurons (Fig. 1a). Quantitative analysis demonstrated high transduction efficacy, with 78.38% and 70.95% of TH+ neurons expressing α-syn at 1-and 3-months, respectively (Fig. 1c). Consistently, GFP expression driven by the same PRSx8 promoter was selectively observed in LC neurons (Supplementary Fig. 1).

The presence of phosphorylated α-syn (α-synP), a key pathological hallmark of protein aggregation was also assessed^23^. While monomeric α-syn was localized in the LC and distributed throughout noradrenergic neuronal projections, α-synP was confined to LC somata, at both 1 and 3 months (Fig. 1b). At 1 month post-injection, 48.48% of TH+ neurons were α-synP+, increasing to 66.22% at 3 months. Moreover, 66.50% of α-syn–expressing neurons were phosphorylated at 1 month, compared to 82.83% at 3 months (Fig. 1d). Altogether, these findings demonstrate that AAV-PRSx8-hα-syn drives selective and sustained α-syn expression in LC noradrenergic neurons, leading to its phosphorylation and providing a robust model to investigate early pathological events associated with α-syn accumulation in this nucleus.

**Fig. 2:**
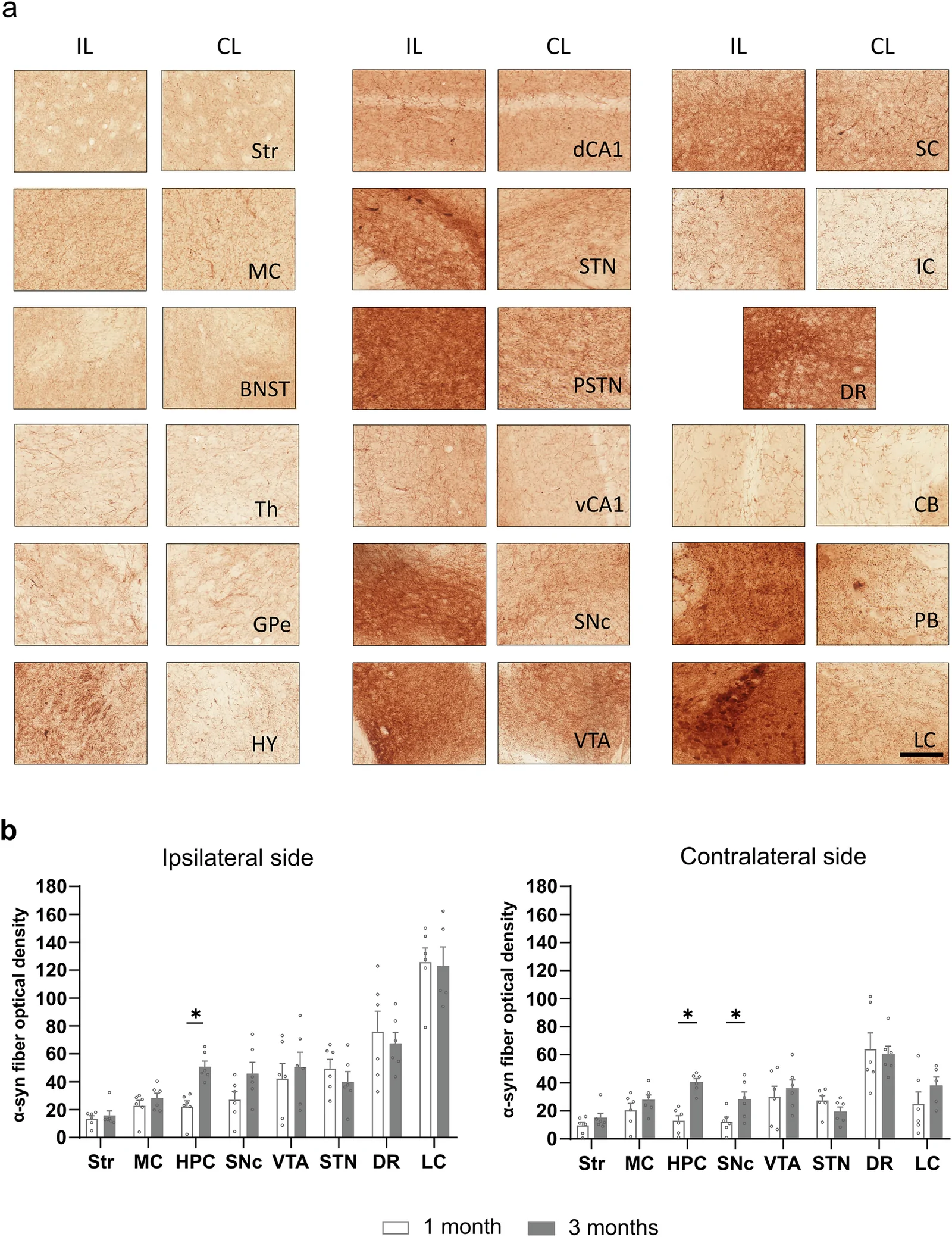
Α-syn expression in unilaterally injected brains. **a** Representative images of ipsilateral and contralateral sides in 1-month α-syn animals following unilateral injection. Scale bar: 100 μm. **b** Α-syn fibre density at 1 and 3 months. *p < 0.05, Student’s *t* test. Str striatum, MC Motor cortex, BNST Bed nucleus of the stria terminalis; Th thalamus, GPe external globus pallidus, HY hypothalamus, dCA1 dorsal CA1, STN subthalamic nucleus, PSTN parasubthalamic nucleus, vCA1 ventral CA1, SNc Substantia nigra pars compacta, VTA ventral tegmental area, SC superior colliculus, IC inferior colliculus, DR dorsal raphe, C cerebellum, PB parabrachial nucleus, LC locus coeruleus, HPC hippocampus. *N* = 6 animals/group.

To determine whether α-syn overexpression propagates from LC neuronal somata along noradrenergic axons, double immunofluorescence staining was performed using antibodies against noradrenergic transporter (NET) and α-syn. LC projection areas, including the PFC and the CA1 layer of the hippocampus (HPC), exhibited a fibre-like distribution of α-syn staining. These α-syn+ fibres clearly colocalized with NET-labelled LC axons (Fig. 1e). These results indicate that α-syn is localized within noradrenergic fibres originating from the LC, supporting the hypothesis that α-syn is primarily localized within and transported along noradrenergic axons of LC projections. To analyse the distribution of α-syn across LC projection areas, α-syn immunohistochemistry was performed on serial brain sections encompassing the entire brain. At 1 month, α-syn+ fibres were already abundant in LC projection areas associated with PD, such as the SNc, subthalamic nucleus, PFC, HPC, motor cortex, and dorsal raphe (DR) (Fig. 1f).

To further analyse α-syn propagation patterns and interhemispheric spread, 14 male mice received unilateral injections of the viral vector. Brains were analysed at 1 and 3 months post-injection, and both qualitative and quantitative analyses were performed. Already at 1 month, most regions exhibited reduced α-syn+ fibre density on the contralateral side to the injection while certain nuclei such as the HPC and ventral tegmental area showed symmetrical distribution of α-syn across both hemispheres. Notably, the LC contralateral to the injection site displayed less α-syn expression compared with more distal areas, including the DR, SNc, subthalamic nucleus and HPC (Fig. 2a and Supplementary Table 2). Semiquantitative analysis confirmed that the HPC and DR exhibited the highest densities of α-syn expression. By 3 months, increased α-syn optical density was observed in the HPC and SNc, while other regions showed stable expression levels (Fig. 2b). Despite robust LC overexpression, interindividual variability in α-syn levels across different brain regions was evident.

### Α-syn accumulation preserves LC and SNc neurons while affecting projection areas

**Fig. 3:**
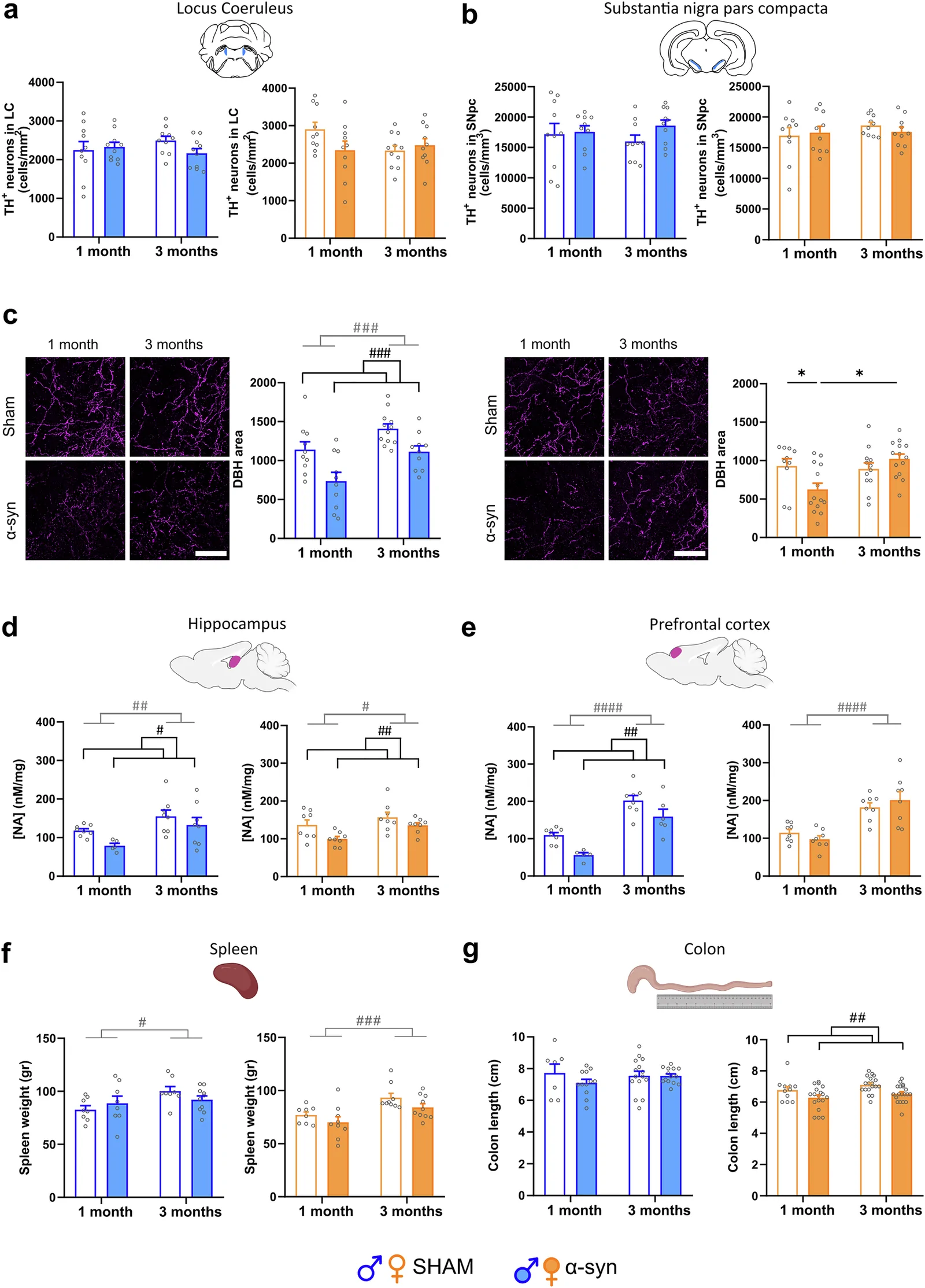
A-syn effects in the brain and peripheral organs in male (blue) and female(orange) mice. **a** TH+ neuron quantification in the locus coeruleus shows no changes between sham and α-syn animals. *N* = 5 animals/group. **b** Stereological analysis of SNc neurons reveals no degeneration. *N* = 5 animals/group. **c** DBH fibre quantification in the hippocampus showed reduction in α-syn mice. Scale bar: 50 μm (×40). *N* = 5–6 animals/group. **d** HPLC analysis revealed decreased noradrenaline levels in the hippocampus and **e** prefrontal cortex in α-syn mice. *N* = 4 animals. **f** No differences were found in spleen weight between sham and α-syn. *N* = 8–10 animals/group. **g** Female α-syn mice exhibited shorter colonl ength. *N* = 7–19 animals/group # *p* < 0.05, ## *p* < 0.01, Two-way ANOVA; *p < 0.05, Tukey’s post hoc test. Created using Biorender.

To determine whether α-syn overexpression induces cell loss, we performed immunofluorescence staining for TH and quantified noradrenergic neurons in the LC. In α-syn mice, no significant reduction in TH+ neurons was observed at either sex or time point (Fig. 3a). Although no significant loss of TH+ neurons was observed in the LC, we note that subtle neuronal loss cannot be completely excluded given the limited sample size. Given the central role of the SNc in PD pathology, we also assessed dopaminergic neuron density in this region using stereological methods. No significant differences were observed between sham and α-syn mice, regardless of sex or time point (Fig. 3b). To investigate whether α-syn expression impacts noradrenergic terminals, we quantified DBH+ fibres in the HPC. Our results showed a significant decrease in DBH+ fibre density in the HPC of male α-syn animals, with a reduction also evident at 1 month in α-syn females (Fig. 3c).

Next, NA levels were measured via high-performance liquid chromatography (HPLC) analysis in the HPC, PFC and general cortical region. In HPC, male α-syn animals displayed a significant reduction in NA levels compared to sham in both sexes (Fig. 3d). In the PFC, NA levels were also reduced in males but not in females (Fig. 3e). This trend was also observed in general cortical area, where α-syn males showed reduced NA concentrations, whereas in females this difference was not detected (Supplementary Fig. 2a). Given that serotonin (5-HT) alterations are also implicated in the prodromal phase of PD and that the LC modulates serotonergic transmission^24–26^, we further analysed 5-HT levels by HPLC. In the HPC, 5-HT was reduced only in α-syn males but not in α-syn females (Supplementary Fig. 2b). In the PFC, both sexes exhibited a significant reduction in 5-HT levels in the α-syn group (Supplementary Fig. 2c). In contrast, in the cortex, 5-HT levels remained unaffected in both sexes (Supplementary Fig. 2d). Striatal DA levels were also measured by HPLC, revealing no differences between sham and α-syn animals (Supplementary Fig. 2e). Likewise, TH⁺ fibre quantification in the striatum showed no differences between groups (Supplementary Fig. 2f). These findings indicate that α-syn overexpression disrupts NA and 5-HT homoeostasis in LC-projection areas in a region and sex-dependent manner while DA levels remain intact, as it has been suggested by others^27^.

To explore potential systemic alterations linked to the α-syn model, we assessed spleen weight and colon length as indicators of systemic inflammation and gut-brain axis dysfunction, respectively. Spleen weight remained unchanged in α-syn animals, but an age-related increase was observed at 3 months (Fig. 3f). Regarding colon measurements, while male α-syn mice showed no differences in colon length, female α-syn mice exhibited a significant reduction compared to sham animals (Fig. 3g). These changes were not accompanied by α-syn detection or morphological modifications in the colon tissue, nor by TNF increases in the serum (Supplementary Fig. 3).

### Sex-dependent functional differences in the LC in the α-syn model

**Fig. 4:**
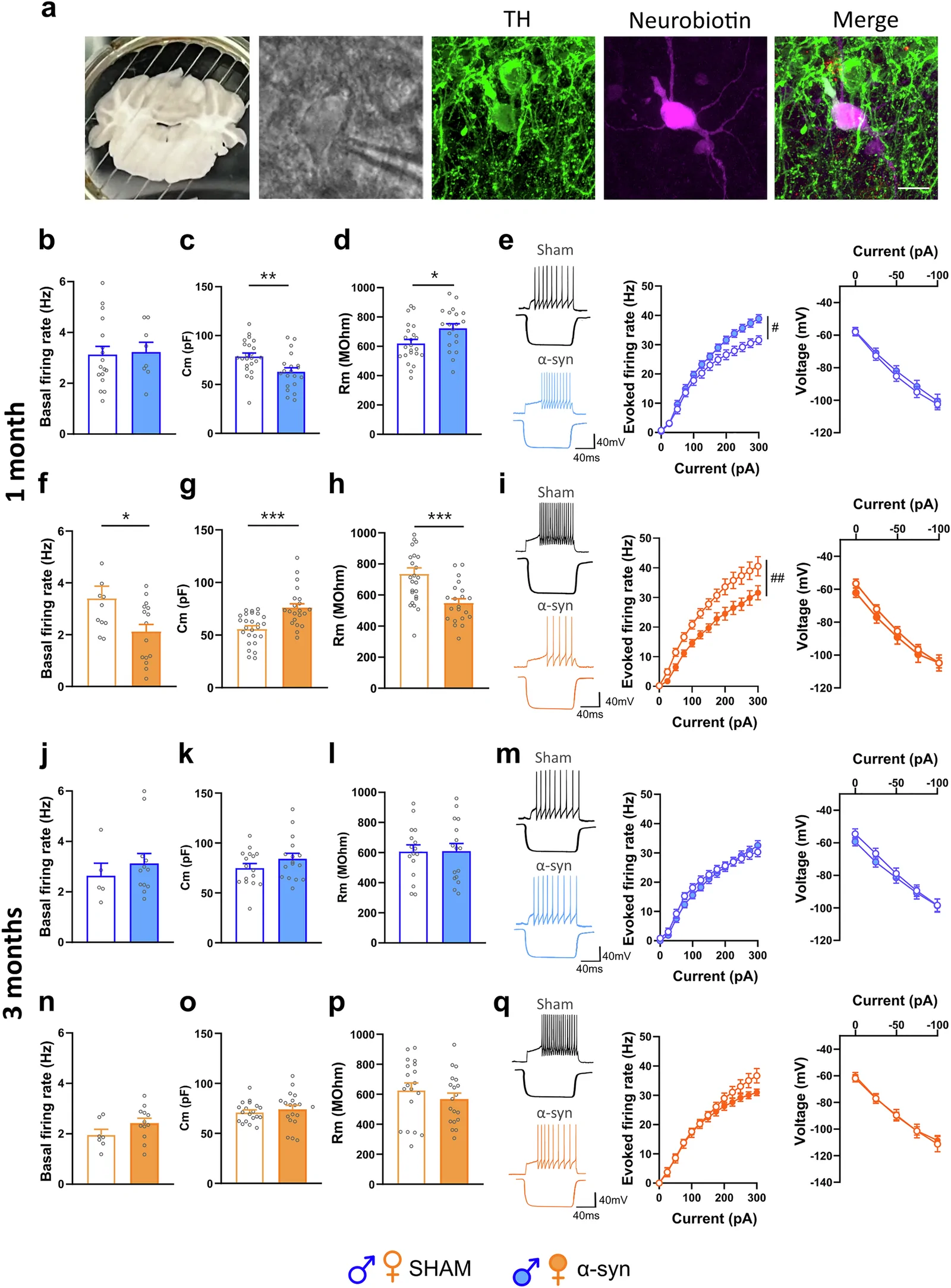
Ex vivo patch clamp recordings of locus coeruleus neurons overexpressingα-syn in male (blue) and female (orange) mice. **a** Representative images of the brain slice and TH+ neurons recorded neurons in the locus coeruleus. **b**–**e** A-syn males exhibited decreased capacitance and increased excitability at 1 month. **f-i** Α-syn females showed reduced spontaneous firing frequency, increased capacitance and decreased excitability 1 month after injection. **j–q** No differences were observed in basal firing rate, intrinsic properties, or excitability between sham (receiving scrambled virus) and α-syn mice at 3 months. *N* = 6 animals/group. Student’s *T* test *p < 0.5, **p < 0.01, ***p < 0.001; Two-way ANOVA # *p* < 0.05, ## *p* < 0.01.

To investigate whether α-syn overexpression induces functional alterations in LC neurons, we conducted patch-clamp recordings (Fig. 4a) at 1 and 3 months post-injection.

The results at 1 month revealed sex-dependent alterations in basal firing rates, passive membrane properties, and excitability. In cell-attached recordings, basal firing rates did not differ in male α-syn mice compared to sham animals (sham 3.13 Hz, α-syn 3.22 Hz) (Fig. 4b). However, female α-syn mice exhibited significantly reduced basal firing rate compared to sham animals (sham 3.41 Hz, α-syn 2.13 Hz) (Fig. 4f). Passive membrane properties also varied among sexes. In males, α-syn expression induced a decrease in membrane capacitance and an increased membrane resistance (Fig. 4c, d). In contrast, female α-syn mice exhibited the opposite effect, with increased membrane capacitance and decreased membrane resistance (Fig. 4g, h). Voltage-current curves highlighted further sex-dependent differences. While α-syn males showed increased excitability with higher evoked firing rates during depolarizing steps (Fig. 4e), female α-syn mice exhibited reduced evoked firing rates (Fig. 4i). Regarding action potential (AP) properties, no relevant significant changes were detected (Supplementary Table 3 and Supplementary Table 4). At 3 months post-injection, none of these electrophysiological alterations were observed. Basal firing rates, passive membrane properties, voltage-current relationships, and AP properties were similar between α-syn and sham animals in both sexes (Fig. 4j-q).

Together, these findings indicate that α-syn expression induces transient, sex-dependent alterations in LC electrophysiology, with functional differences evident at 1 month but not sustained at later stages.

### A-syn overexpression induces cognitive deficits and altered affective behaviour

To determine whether the observed LC functional alterations translate into behavioural outcomes, we next assessed motor, affective, and cognitive functions in α-syn mice.

**Fig. 5:**
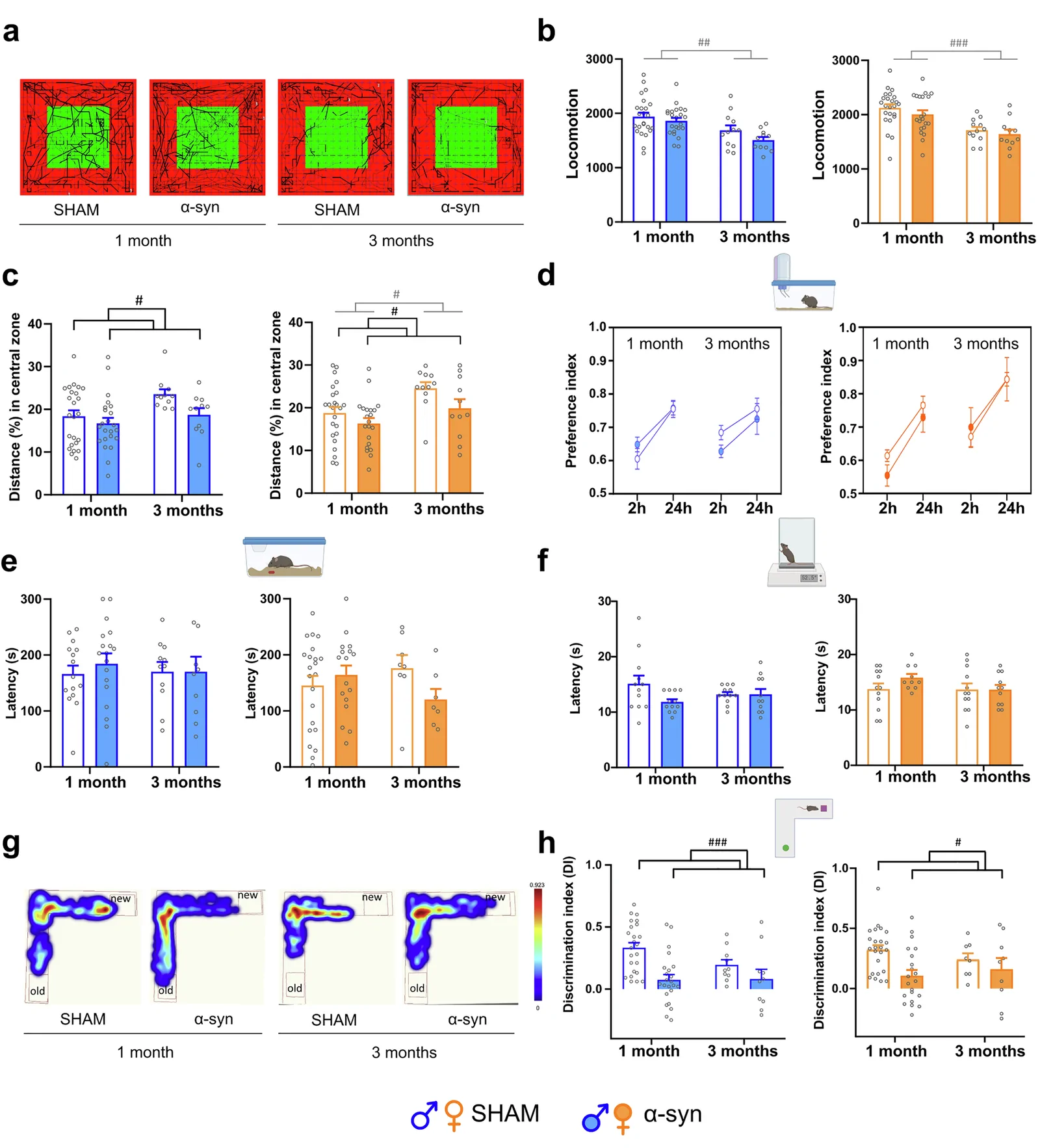
Behavioural evaluation of the α-syn model in male (blue) and female (orange) mice. **a** Representative images of open field performance. **b** No motor differences were observed between sham and α-syn mice. **c** Α-syn overexpression reduced the distance percentage in the centre of the arena, indicating anxiety-like behaviour in both sexes. No differences were found in anhedonia (**d**), olfaction (**e**), or nociception (**f**). **g** Heat maps of exploratory behaviour in the novel object recognition test. **h** Discrimination index was significantly reduced in α-syn males. *N* = 10–24 animals/group. # *p* < 0.05, ### *p* <  0.001, Two-way ANOVA. Created using Biorender.

Our results suggested preserved motor function with no significant differences in locomotion (Fig. 5a, b), general activity or stereotypic movements (Supplementary Fig. 3a–d). This finding aligns with the absence of cell loss in the SNc and is consistent with the premotor phase of PD, where motor symptoms are absent.

Given the relevance of NA in neuropsychiatric symptoms appearance in PD^13–15^, we evaluated anxiety-like behaviour and anhedonia. In the open field (OF) test, α-syn mice of both sexes travelled significantly less in the centre of the arena (Fig. 5c), indicating reduced innate-emotional behaviour. This was further supported by the dark and light box test were female α-syn mice exhibited shorter latencies to enter the dark zone whereas no significant differences were observed in males (Supplementary Fig. 4e-h). In contrast, no significant differences were detected in the Elevated Plus Maze (EPM) (Supplementary Fig. 4i–l), indicating that anxiety-like alterations were not consistently observed across all behavioural paradigms. Sucrose preference did not differ between α-syn and sham animals, indicating no clear signs of anhedonia (Fig. 5d). Olfactory function was evaluated using the buried food task, revealing no significant differences between α-syn and sham animals (Fig. 5e). Similarly, nociceptive responses in the hot plate test showed no overall differences (Fig. 5f). Interestingly, cognitive deficits were evident in the α-syn mice, as shown in the novel object recognition (NOR) task. Α-syn animals displayed robust lower discrimination indices, regardless of sex (Fig. 5g, h).

Overall, behavioural evaluation revealed robust cognitive deficits and mild and context-dependent alterations on innate-emotional behaviours, which could reflect a potential anxiety phenotype in male and female α-syn mice. Importantly, these behavioural deficits were persistent and remained 3 months after AAV injection.

## Discussion

The contribution of noradrenergic dysfunction to PD pathogenesis remains unclear, largely due to the lack of models that selectively target the LC. Here, we described a novel mouse model that overexpresses human α-syn in LC neurons, reproducing key prodromal features including neuronal α-syn accumulation, axonal pathology, monoaminergic disruption, sex-dependent electrophysiological alterations and cognitive deficits without overt neuronal loss. This model uniquely isolates LC-driven pathology, providing a platform to dissect circuit-specific mechanisms and translational interventions.

Using the PRSx8 promoter, we achieved selective human α-syn expression in TH + LC neurons with high efficiency^28^. Phosphorylated α-syn, a hallmark of early aggregation and Lewy body formation^23^, was detected in somata as early as 1 month after AAV- PRSx8-hasynWT injection, while monomeric α-syn propagated extensively along LC axons. Consistent with previous studies using similar α-syn carrying-viral vectors^27,29^, soluble α-syn species were detected in projection areas largely colocalizing with NET+ fibres, suggesting α-syn axonal transport^30–32^. The presence of α-syn in some regions from the contralateral hemisphere, as the HPC, aligns with the bilateral innervation of LC projections to certain areas^27^ and with the evidence that commissural pathways may facilitate the interhemispheric communication^2^. Supporting this, animal models have provided compelling evidence that α-syn can propagate to anatomically connected brain regions^33^.

In our model, despite robust α-syn accumulation, LC and SNc neuronal loss was absent, consistent with other models targeting the LC^22,27^. This neuronal survival also agrees with the clinical and preclinical observations that LC neurons have long resilience and can survive years despite α-syn pathology^34,35^. Our mice showed reduced DBH+ fibres and decreased NA in LC projection areas, indicating a “dying-back” process where axons degenerate prior to soma loss^15,36^. This pattern is consistent with previous reports indicating that noradrenergic projections are particularly vulnerable, whereas LC neuronal cell bodies appear comparatively resistant to multiple insults. Notably, recent imaging studies in PD patients further suggest that LC terminal loss may precede neuronal degeneration within the LC^37^. Similar findings have been reported in animal models of α-syn overexpression, including SNc-targeted α-syn models^38^, DBH-hSNCA mice and toxic-based models of noradrenergic damage using DSP-4, neuromelanin, 6-OHDA or MPTP^21,22,35,39,40^. Together, these studies support the concept of selective axonopathy in α-synucleinopathies^41^.

In addition to NA content loss, serotonergic transmission was also affected in our α-syn model, as happens in patients^42^ and some mouse models of the disease^43^. The fact that the LC influences DR activity has been well described in the literature. The LC extensively innervates the DR^44^, modulates its excitability^25^ and compromises 5-HT release^24^. This interplay highlights the LC as a hub integrating monoaminergic balance across cortical and limbic targets.

In parallel to neuroanatomical changes, patients with PD often show gut inflammation or dysbiosis^45^, supporting bidirectional communication along the gut-brain axis^2^. In our study, enteric alterations were subtle but sex-specific. Colon length was reduced in α-syn females without accompanying morphological changes. Consistently, the absence of changes in spleen weight suggests minimal systemic inflammatory involvement^46^, which was confirmed by unmodified serum TNF levels. One potential mechanism to explain our results is that central noradrenergic dysfunction modulates colonic motility and tone independently of the presence of α-syn. The LC regulates colorectal function via spinal pathways and monoaminergic receptors, and noradrenergic imbalance disrupts colonic transit^47^. Although, in general, female tend to develop less severe motor pathology than males in α-syn parkinsonian models^48^, emerging evidence indicates increased vulnerability to specific enteric alterations in PD patients^49^ and experimental models. For instance, rotenone-induced parkinsonian females showed greater myenteric α-syn accumulation and more pronounced neuronal changes than males^50^. Sex shapes gut microbiome responses to stress and diet^51^ and oestrogenic modulation of enteric function may also contribute to this difference^52^.

Some authors suggest that, in the early stages of PD and Alzheimer’s, LC neurons become hyperexcitable due to α-syn overexpression, exhibiting increased firing rates that may precede subsequent hypofunction and degeneration^34^. In our experiments, however, we observed a reduction in firing frequency exclusively in females, with no changes in males. Previous studies overexpressing wild-type human α-syn in the LC of male mice reported no alteration^53^, while other LC-targeted models have shown variable outcomes. For instance, 6-OHDA injections and neuromelanin overexpression increased basal firing and pattern of LC neurons^54,55^, whereas DSP-4 intraperitoneal administrations did not affect tonic activity^21^. Classical PD models targeting the medial forebrain bundle or the SNc, using 6-OHDA^56^, rotenone^57^, MPTP^58^, and preformed fibrils^59^, typically report compensatory reductions in spontaneous LC and SNc neuron activity. Importantly, most of these studies were conducted in males or pooled sexes^21,55^, potentially masking sex-specific responses. The mechanisms underlying the reduced firing activity in α-syn females remain unclear and warrant further investigation. Our AP analyses revealed no changes in afterhyperpolarization or other electrophysiological parameters, suggesting that major ion channel dysfunction is unlikely. Nonetheless, subtle changes in L- and T-type Ca²⁺ channels^60^ and enhanced GABAergic inhibition, cannot be ruled out^61^.

Beyond firing frequency, we also found sex-dependent differences in intrinsic membrane properties. Α-syn females displayed higher membrane capacitance, whereas α-syn males showed lower capacitance relative to their respective controls. These opposing effects were accompanied by distinct excitability profiles where neurons from males exhibited hyperexcitability and in females were hypoexcitable. The increased excitability observed in α-syn males may result from smaller neuronal size, as indicated by their reduced capacitance^62^. This may be further exacerbated by the more pronounced monoamine depletion found in males, which likely reflects a greater compromise of LC projections. Although SK-channel dysfunction has been implicated in α-syn induced hyperexcitability^53^, our data do not support this mechanism, as afterhyperpolarization amplitude was unchanged. Alterations in receptor signalling might also contribute; for example, CB1 receptor deletion in the LC selectively increases excitability in male neurons while leaving females unaffected^63^. In contrast, the hypoexcitability observed in females may relate to increased membrane capacitance, supporting the idea that α-syn overexpression drives distinct morphological and functional adaptations in each sex.

Interestingly, the transient increase in LC neuronal excitability observed at 1 month, followed by normalization at 3 months, likely reflects a homoeostatic compensation that restores somatic firing without resolving circuit dysfunction^53^. Some neurochemical and histological results were partially recovered at 3 months, especially in females, while behavioural deficits may reflect permanent alterations in projection regions. Similar adaptations have been observed in toxin-based models. For example, administration of DSP-4 induces a transient disruption of LC neuronal activity followed by compensatory sprouting of surviving axons, increased neurotransmitter synthesis and release from residual terminals^64–66^. More recently, a work performed in mice overexpressing A53T α-syn in the SNc, demonstrated early striatal long-term potentiation deficits that partially recovered via structural dendritic spine remodelling despite persistent dopaminergic loss^67^. Future studies examining neuron activity and synaptic plasticity in projection areas, including hippocampal neurons, will be important to link persistent behavioural impairments with circuit-level dysfunction.

Our electrophysiological findings also highlight the importance of considering sex as a biological variable when studying LC physiology, both under normal and pathological conditions. Sex dimorphism is a well-established feature of the LC in control mice, with females exhibiting greater neuronal number, enhanced dendritic complexity, and distinct electrophysiological and molecular profiles^68–70^. The sex-specific patterns of LC excitability observed in our study, align with results from a model of chronic constriction neuropathic pain, where LC hyperexcitability was observed exclusively in males while females exhibited reduced neuronal excitability^71^. Similar to our results, animals of both sexes performed equally in the NOR test, highlighting that divergent somatic adaptations may share common downstream consequences at the circuit and behavioural levels.

Given the involvement of the LC in PD symptomatology, we next assessed whether its selective α-syn overexpression could lead to behavioural alterations, following the anatomical and functional analyses. Motor impairment, a hallmark of PD, results from the degeneration of dopaminergic neurons in the SNc, which can be exacerbated by LC dysfunction^72^. In our model, motor function, assessed in the OF, was preserved, consistent with the anatomical findings showing that dopaminergic neuron number in the SNc and DA striatal levels remained unaffected despite elevated α-syn expression. This observation aligns with previous studies employing LC-targeted α-syn expression or DSP-4 lesions, where also locomotor activity was unaltered^22,55^.

Other non-motor behaviours were similarly unchanged, as anhedonic behaviour, olfaction or pain. Although reductions in 5-HT and NA levels were observed, a neurochemical feature often linked to major depressive disorder^73^, these changes did not translate into behavioural deficits. We acknowledge that behavioural despair paradigms, such as the tail suspension test and the forced swim test, are generally more sensitive for detecting depression-like phenotypes, particularly in mild or prodromal models, and may reveal alterations not captured by sucrose preference alone^74^. Methodological factors, such the sucrose concentration^75^ or the absence of deprivation protocols, may have contributed to this outcome. Accordingly, these approaches will be considered in future studies. Likewise, no olfactory impairment was detected, possibly influenced by the lack of food deprivation^76^ or by the limited time window for α-syn propagation from the LC to the olfactory bulbs^77,78^. Finally, thermal nociception thresholds remained unchanged, in agreement with prior evidence suggesting that heat sensitivity is often spared in PD^79^, and that noradrenergic dysfunction may selectively influence other pain modalities^80^.

In contrast to the preserved motor and non-motor functions described above, cognitive performance and, to a lesser extent, innate emotional behaviour were affected across the evaluated time points. Anxiety is a common non-motor symptom of PD, affecting up to 60% of patients and often manifesting as generalized anxiety, panic attacks, or social phobia^81^. In our model, α-syn animals displayed subtle and context-dependent alterations on innate emotional behaviour, which could reflect a potential anxiety phenotype. Importantly, different behavioural assays may produce divergent results, as they assess distinct aspects of anxiety-like behaviour^82–84^. This phenotype may be linked to the decreased NA levels observed in the PFC of α-syn animals, a region critically involved in anxiety regulation. Supporting this interpretation, inhibition of the LC-PFC pathway has been shown to increase anxiety-like behaviour in stressed rodents^85^. These observations align with anxiety phenotypes reported in neuromelanin transgenic models^86^ and other α-syn models, including DBH-hSNCA mice^22^, raphe-targeted overexpression models^29^, and transgenic lines^43^.

Our animals also exhibited robust cognitive impairment, consistent with clinical observations in PD. Cognitive decline affects up to 83% of PD patients^87^ and, although traditionally linked to cholinergic and dopaminergic dysfunction, growing evidence points to noradrenergic degeneration, particularly LC dysfunction, as a major contributor^88^. In our α-syn model, both male and female animals exhibited significant cognitive deficits in the NOR task. The 24-h retention interval employed strongly engages hippocampal-dependent long-term memory^89^, suggesting that the observed deficits reflect hippocampal rather than perirhinal dysfunction. Consistent with this, NA levels were reduced in both the HPC and PFC, areas densely innervated by the LC^90^, which co-releases NA and DA^91^ and plays a central role in novelty detection, attention, and cognitive flexibility^92–94^. Moreover, α-syn expression was evident in noradrenergic hippocampal projections, suggesting direct involvement of LC-derived fibres in local circuit alterations. Given that the LC bilaterally innervates the HPC, even restricted unilateral α-syn overexpression is likely to compromise hippocampal integrity, a notion supported by comparable cognitive impairments described in other α-syn models^95,96^. Collectively, these data indicate that selective α-syn overexpression in LC neurons is sufficient to trigger emotional and cognitive deficits, while sparing motor performance. This pattern mirrors the pre-motor phase of PD, in which early noradrenergic dysfunction contributes to emotional and cognitive disturbances preceding overt motor decline.

In conclusion, selective overexpression of α-syn in noradrenergic LC neurons recapitulates key features seen in early PD, including somatic α-syn accumulation, widespread axonal distribution, and monoaminergic dysfunction without overt neuronal loss. Our data support that axonal degeneration precedes somatic compromise, together with a functional dissociation between cell bodies and projection sites. In addition, the transient alterations observed in some parameters support the resilience of LC neurons, in contrast to other regions such as the SNc. Behaviourally, the model recapitulates mild emotional alterations and marked cognitive impairment in both sexes, while preserving motor function and dopaminergic integrity, mirroring early PD progression. The presence of sex-specific alterations in neuronal excitability underscores the importance of considering sex as a biological variable in the disease. Overall, these findings support a central role for the LC in α-syn-mediated circuit dysfunction contributing to non-motor symptoms, and highlight its potential as a therapeutic target.

## Methods

### Animals

131 male and 117 female C57BL/6J mice (Envigo, Spain), 8 weeks old at the beginning of the experiment, were used in this study. Animals were housed in groups of six under standard laboratory conditions (22 ± 1 °C, 55 ± 5% humidity, and a 12:12 h light/dark cycle) with *ad libitum* access to food and water. All procedures complied with ARRIVE guidelines, EU Directive 2010/63/EU, and Spanish regulations (RD 53/2013), and were approved by the Local Committee for Animal Experimentation at the University of the Basque Country (#M30-2019-220, #M20-2019-219, #M20-2022-215 and #M30-2022-220).

### Experimental design

**Fig. 6:**
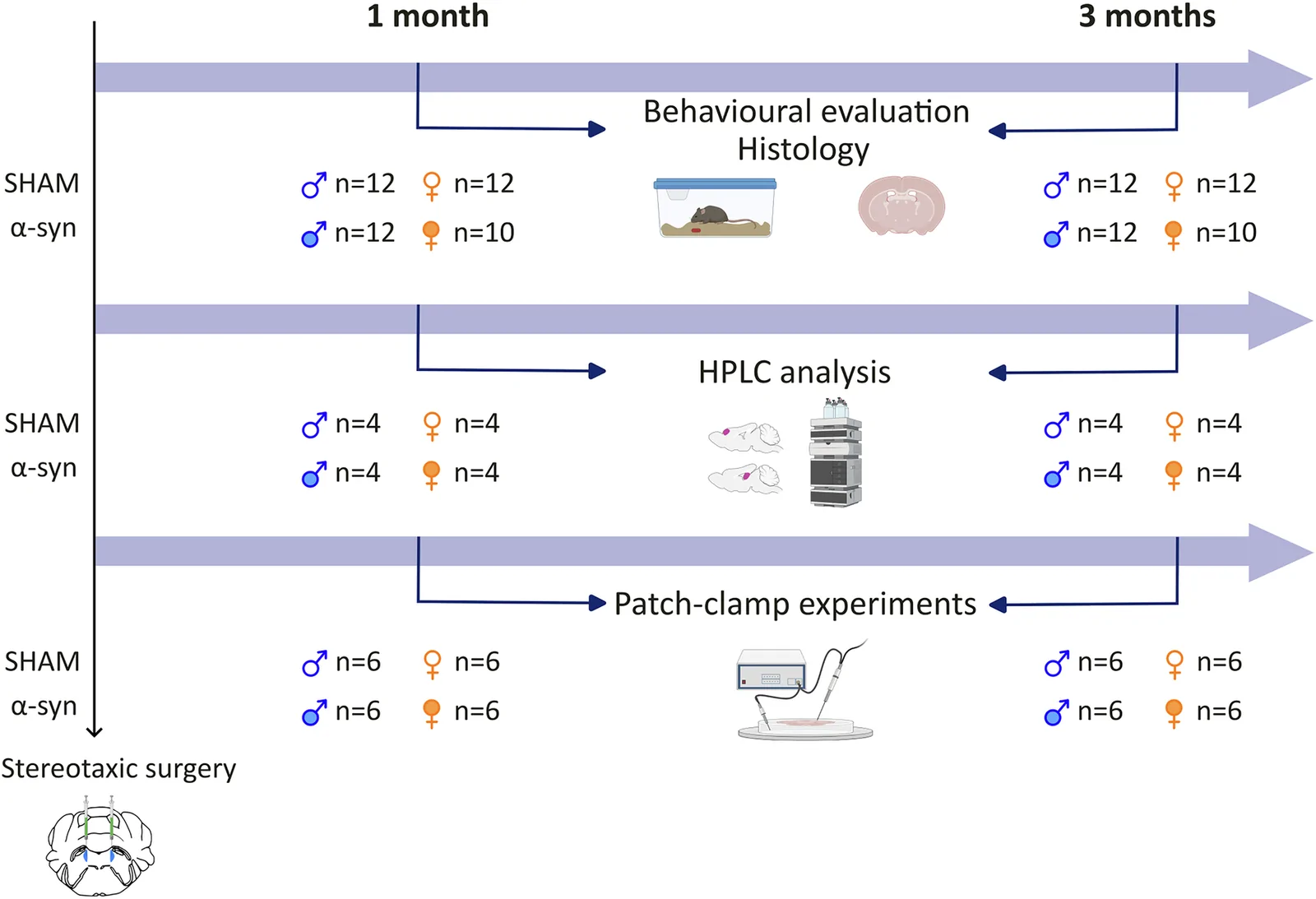
Experimental design. Stereotaxic surgery was performed in male and female mice to inject the viral vector. After establishing the α -syn model, behavioural tests, histological analyses, HPLC measurements, and ex vivo electrophysiological recordings were conducted at 1 and 3 months post-surgery. Created using Biorender.

At the beginning of the experiments, animals were stereotaxically injected with AAV9 viral vectors to express either human α-syn, GFP, or a control vector lacking a transgene. Behavioural, histological, neurochemical, and electrophysiological assessments were performed at 1 and 3 months post-injection (Fig. 6). Sample sizes were determined based on previous studies using similar experimental approaches. For behavioural experiments, the order of testing was randomized using Excel’s RAND() function, assigning one random number per animal. The experimenter was blinded to group allocation during data collection and analysis.

### Stereotaxic surgery

Mice received unilateral or bilateral injections into the LC of viral vectors encoding hSNCA under a DBH-specific promoter: GFP (AAV9-MCS-PRSx8-GFP-WPRE), scrambled control (AAV9-MCS-PRSx8-WPRE), or α-syn (AAV9-MCS-PRSx8-h-α-synWT-WPRE). A volume of 0.5 µL per site (1.07 × 10¹³ gcp/mL) was infused. Viral constructs were produced at CIMA (Universidad de Navarra, Spain). Animals were anesthetized with isoflurane (4% induction, 1.5–2% maintenance), placed in a stereotaxic frame and maintained at 33 °C. Eye lubrication and meloxicam (2 mg/kg, s.c.) were administered. Injections targeted the LC (AP − 5.4 mm, ML ± 0.9 mm, DV − 3.5 mm from bregma) at 0.2 µL/min using glass pipettes. The needle was left in place for 5 min post-injection to allow diffusion.

### Immunohistochemical procedures

#### Tissue processing

Animals were deeply anesthetized with ketamine/xylazine (100/10 mg/kg, i.p.) and transcardially perfused with 4% paraformaldehyde (PFA). Brains were post-fixed (24 h, 4% PFA), dehydrated in 30% sucrose and sectioned (40 μm) using a freezing microtome (Microm 440E, Germany). Sections were stored in cryoprotectant solution at –20 °C until further processing. Slices from electrophysiological recordings were processed similarly.

#### Immunohistochemistry with 3’3 Diaminobencidine (DAB)

DAB staining was performed to detect TH and α-syn across brain regions. Free-floating sections were treated with 0.9% H₂O₂ in PBS for 20 min, blocking solution and incubated overnight with primary antibodies (Supplementary Table 1) at room temperature. After washing, sections were incubated with secondary antibodies for 2 h (Supplementary Table 1). Antigen signal was amplified using VectaStain® Elite ABC-HRP Kit 1:100 during 1 h at room temperature. The signal was revealed using DAB and 3% H_2_O_2_. After, sections were mounted, dehydrated in xylene, and coverslipped with DPX.

#### Immunofluorescence staining

Sections were rinsed in PBS, blocked (5% NGS, 0.3% Triton X-100), and incubated overnight at 4 °C with primary antibodies (Supplementary Table 1). After washing, sections were incubated with secondary antibodies, mounted, and coverslipped with Vectashield mounting medium (Vector Labs, Peterborough, UK). For DBH staining, antigen retrieval (2 N HCl, 15 min) was performed prior to standard processing.

#### Quantification and analysis

Noradrenergic neurons of the LC were quantified using TH immunofluorescence, while α-syn and α-synP staining was employed to quantify colocalization with TH+ neurons. TH and α-syn quantification was performed in 5 mice per group using 3 sections per animal (bregma −5.34 to −5.52 mm). Double immunofluorescence staining for TH and α-syn was performed as previously described. Images were acquired using structured illumination (Zeiss Apotome) or confocal microscopy (Zeiss LSM880 Airyscan) and processed with ImageJ/FIJI. Images were acquired at 20× with Z-stacks (0.9 μm step), and for α-synP analysis, 10–13 LC were evaluated per time point using structured illumination. Images were processed by deconvolution and maximum intensity projection. Cell counts and colocalization analyses were performed manually by an experimenter blinded to experimental conditions. A-syn neuron colocalization was expressed as efficacy ((α-syn+TH)/TH) and specificity ((α-syn+TH)/α-syn). For α-synP, colocalization was calculated relative to TH+ neurons and total α-syn+ neurons. Neuronal density was expressed as TH+ cells/mm².

TH+ neurons in the SNc were quantified stereologically using Mercator image analysis system (Explora-Nova, La Rochelle, France) connected to an Olympus BX51 microscope. We used five animals per group and 7 slices from each hemisphere (bregma –2.92 to –3.16 mm). Boundaries of the area of the SNc were traced as an ROI (region of interest) with the 4x objective. The sections were quantified using a counting frame size 50×50μm and a distance of 60 μm × 60 μm between dissectors. The total number of cells was estimated as: N = ΣQ x (1/ssf) x (1/asf) x (1/hsf), where Q represents the actual number of cells counted in a sample, and N is the total cell estimation. In this study, the section sampling fraction (ssf) was 1/3 and volume estimations were obtained using Cavalieri´s method.

Α-syn overexpression was assessed using DAB immunostaining (*n* = 6 for each condition). Sections were captured using high resolution slide scanner Pannoramic MIDI II (3DHistech, Hungary). Optical density was measured in selected brain regions, striatum, cortex, HPC, SNc, ventral tegmental area, subthalamic nucleus, dorsal raphe, and LC, and normalized to background signal within the same section. Similarly, striatal TH fibre density was assessed by DAB staining (6 sections per animal). Images were acquired using an EPSON Perfection V750 Pro scanner. The striatum was manually delineated, and optical density was calculated after background subtraction using the cortex as reference. Mean values per animal were used for statistical analysis. The images were analyzed using FIJI software^97^.

#### Brain tissue NA, 5-HT and DA analysis

Mice were euthanized under ketamine-xylazine anaesthesia (100/10 mg/mL) and brains were rapidly dissected. The HPC, general cortex, PFC and striatum were isolated and frozen until analysis, while caudal regions were processed for α-syn-DAB immunohistochemistry to confirm LC overexpression.

Samples were weighed and homogenized in 1 N perchloric acid containing 100 μM EDTA (15 μL/mg tissue) using an Ultra-Turrax® T-10 homogenizer, followed by centrifugation (15 min, 4 °C, 20,817 × *g*). The supernatant was filtered (Costar Spin_XTM 0.22 μm, Sigma-Aldrich) by a second centrifugation (5 min, 4 °C, 1000 × *g*). NA, 5-HT and DA levels were measured using HPLC with electrochemical detection (VT-03, Antec Scientific) at 0.3 V, with an injection volume of 35 μL. Separation was achieved with a C18 column (ALF-215, 2.1 × 150 mm, Antec Scientific) and a mobile phase containing 50 mM phosphoric acid, 0.1 mM EDTA, 8 mM NaCl, 500 mg/L sodium octyl sulphate, and 12% methanol (pH 6.0, 0.2 ml/min flow rate). Chromatographic data were analysed using Chemstation plus software, and monoamine concentrations were determined via linear regression of standard curves (0.5–400 nM). Monoamine levels were normalized to tissue weight and are expressed as nM/mg.

#### Colon histology, immunohistochemistry and ELISA

Colon tissue was collected, fixed in 4% PFA (72 h), paraffin-embedded, sectioned (4 μm) and mounted on glass slides. Sections were deparaffinized (xylene, 10 min), rehydrated in acetone and endogenous peroxidase activity was blocked (methanol containing 4% H_2_O_2_, 20 min). Antigen retrieval was performed using trypsin solution (10 min, 37 °C), following by non-specific binding sites blocking. Sections were incubated overnight at 4 °C with the primary antibody (Supplementary Table 1) in a humidified chamber. For immunohistochemistry, sections were incubated with a biotinylated secondary antibody (1 h, room temperature), followed by avidin-peroxidase complex amplification (45 min) and visualization with DAB. Sections were counterstained with Harris Hematoxylin (1 min), dehydrated and coverslipped using DPX mounting medium. For immunofluorescence, sections were incubated with fluorescent secondary antibodies (Supplementary Table 1) in the dark, counterstained with Hoechst, and coverslipped using Mowiol.

For the analysis of serum TNF levels, blood was collected by cardiac puncture under deep ketamine/xylazine (100/10 mg/kg, respectively) into serum collection tubes (without anticoagulant) and allowed to clot at room temperature. Samples were centrifugation two times and stored at −80 °C. Serum TNF levels were determined using a mouse TNF ELISA set II (BD OptEIA), according to the manufacturer’s instructions.

#### Spleen weight and colon measurement

The spleen and colon were removed for measurement and weighing after sacrificing the animal, when perfusion was not performed.

#### Patch-clamp electrophysiology

Mice were anesthetized with 5% isoflurane, then decapitated, and their brains were rapidly removed and kept in cold sucrose-based artificial cerebrospinal fluid (ACSF) (2.5 KCl, 1.25 NaH₂PO₄, 2.5 KCl, 0.5 CaCl₂, 10 MgSO₄, 10 glucose, 230 sucrose). Coronal sections (220 μm) containing the LC were cut using a vibratome (Microm HM 650 V) in cold. Slices were incubated in oxygenated ACSF at 35 °C for at least 30 min before recording. The ACSF had the following composition (in mM): 126 NaCl, 1.25 NaH₂PO₄, 2.5 KCl, 2 CaCl₂, 2 MgSO₄, 10 glucose, 26 NaHCO₃, 1 sodium pyruvate, and 4.9 L-glutathione reduced. The solution was equilibrated with 95% O₂ and 5% CO₂, maintaining a pH of 7.3–7.4. Slices were transferred into the recording chamber and perfused continuously with oxygenated modified ACSF (126 NaCl mM, 1.25 NaH₂PO₄ mM, 3 KCl, 1.6 CaCl₂ mM, 1.5 MgSO₄ mM, 10 glucose mM, 26 NaHCO3 mM) at 32–34 °C. LC neurons were identified using infrared differential interference contrast microscopy (Eclipse E600FN, Nikon). Patch-clamp recordings were performed using borosilicate glass electrodes (3–6 MΩ) filled with an internal solution containing (in mM): 135 K-Gluconate, 10 HEPES, 3.8 NaCl, 1 MgCl₂, 0.1 EGTA, 2 Mg-5ATP and 0.4 Na_2_-GTP (pH 7.2, 290 mOsm). Neurobiotin (1%) was added to the internal solution on the day of the experiment and later subjected to immunohistochemical staining, in which streptavidin was used together with TH immunodetection.

Signals were recorded using an Axopatch 200B amplifier and Digidata 1322 A digitizer (Molecular Devices) controlled by Clampex 10.3. Voltage-clamp recordings were low-pass filtered at 2 kHz and sampled at 10 kHz, and current-clamp recordings were filtered at 5 kHz and sampled at 20 kHz. Junction potential was 15 mV and was not corrected. Spontaneous firing rates were recorded in the cell-attached configuration with neurons held at −60 mV. LC neurons were identified by a resting inwardly rectifying potassium conductance, measured by stepping the membrane potential from -40 to -120 mV in -10 mV steps. Passive membrane properties were assessed by injecting -5 mV steps to measure capacitance and resistance. In current-clamp mode, current pulses (-300 to +300 pA in 25 pA steps) were injected to assess evoked firing rates and generate current-voltage curves. AP properties were analysed from the first sweep without current injection. Data were analysed off-line using Clampfit 10.7 software (Molecular Devices). Neurons were treated as individual observations for statistical analysis.

### Behavioural assessment

#### Open field test

Spontaneous locomotor activity was measured in an open field arena (44 ×44 x 35 cm) using Actitrack software (Panlab, Spain)^98^. Each animal was placed in the centre of the arena, and its activity was recorded for a period of 10 min.

#### Elevated plus maze

The elevated plus maze test was employed to evaluate anxiety-like behaviour in mice^99^. The apparatus consisted of two open arms (66 × 5.5 cm) and two closed arms (66 × 5.5 cm) arranged in a cross shape on a platform elevated 49 cm above the ground. Each mouse was placed in the centre of the maze facing an open arm and allowed to explore the maze freely for 5 min.

#### Dark and light box

To assess anxiety-like behaviour using a more aversive test, the dark and light box test was conducted^100^. The box (Panlab, Spain) consists of an arena with two separate zones (26 × 26 cm). One side maintains a dim red light and a black floor, while the other side has a brighter light with a white floor. Each mouse was placed in the centre of the illuminated area facing away from the dark zone and allowed to explore for 5 min.

#### Sucrose preference test

To assess anhedonia in mice, the sucrose consumption test was employed. Mice were first habituated to drink from two bottles to avoid any side bias before conducting the test. During the test phase, one bottle was filled with water, and the other with a 3% sucrose solution. The placement of these bottles was randomized to prevent positional bias. The mice’s consumption of each liquid was measured by weighing the bottles at 2 and 24 h.

#### Buried food task

Olfactory function was evaluated using the buried food task without prior food deprivation^101^. Mice were individually housed and familiarized with the scented pellet (Versele-Laga’s Complete Crock®, cheese or apple flavours) by placing the pellet in their cages for 72 h to ensure they consumed it. On the first day, the mouse was allowed to explore for 5 min the test cage containing only sawdust (3 cm deep). On the second day, the scented pellet was buried in the sawdust, and the animal was given 5 min to explore.

#### Hot plate

The hot plate test was employed to evaluate hyperalgesia in mice^102^. Each mouse was placed on a metallic surface maintained at 52.5 °C, surrounded by a 40 cm high plexiglass wall (Digital DS-37 Socrel model; Milan, Italy). The latency to show signs of pain, such as paw licking or jumping, was recorded. A maximum time limit of 45 s was set to avoid any tissue damage.

#### Novel object recognition test

Cognitive performance was assessed using the novel object recognition test in an L-shaped maze (8 × 37 × 14.5 cm)^103^. The task was conducted over three consecutive days (9 min/session): habituation (empty arena), acquisition (two identical objects), and test (one familiar and one novel object). Animals were placed facing the wall at the start of each session. Exploration was defined as orientation towards the object with the nose within 2 cm and was quantified using Behaviour Scoring Panel software (© 2008 by A. DUBREUCQ, version 3.0 beta) with heat maps generated using EthoVision. Memory performance was evaluated by calculating the discrimination index: (Tnew−Told) / (Tnew+Told), where Tnew represents the time spent exploring the novel object, and Told represents the time spent exploring the familiar object.

#### Statistical analysis

Data were analysed using GraphPad v8.0.1. software with statistical significance set at *p* < 0.05. Normality was assessed using the Shapiro–Wilk test, and outliers were identified prior to analysis. Results are presented as mean ± SEM. Comparisons involving a single variable were performed using Student’s *t* test. For experiments including treatment (sham vs α-syn) and time, two-way ANOVA was applied, followed by Tukey’s post hoc test when significant interactions were detected. Sample sizes were determined based on previous studies and preliminary data to ensure adequate statistical power.

## Supplementary information

Supplementary material

## Data Availability

The datasets generated and analysed during the current study are available in Zenodo repository, https://doi.org/10.5281/zenodo.19865488. Data are available on request to the corresponding author.
