# Supplementary material for: Locus coeruleus α-synuclein overexpression induces prodromal Parkinsonian features in mice
Source: NPJ Parkinsons Dis. 2026 Jun 8;12:225. doi: 10.1038/s41531-026-01420-w (PMC13583003; doi:10.1038/s41531-026-01420-w)
Supplement: Supplementary file 1 [file 41531_2026_1420_MOESM1_ESM.pdf]

Supplementary Figures

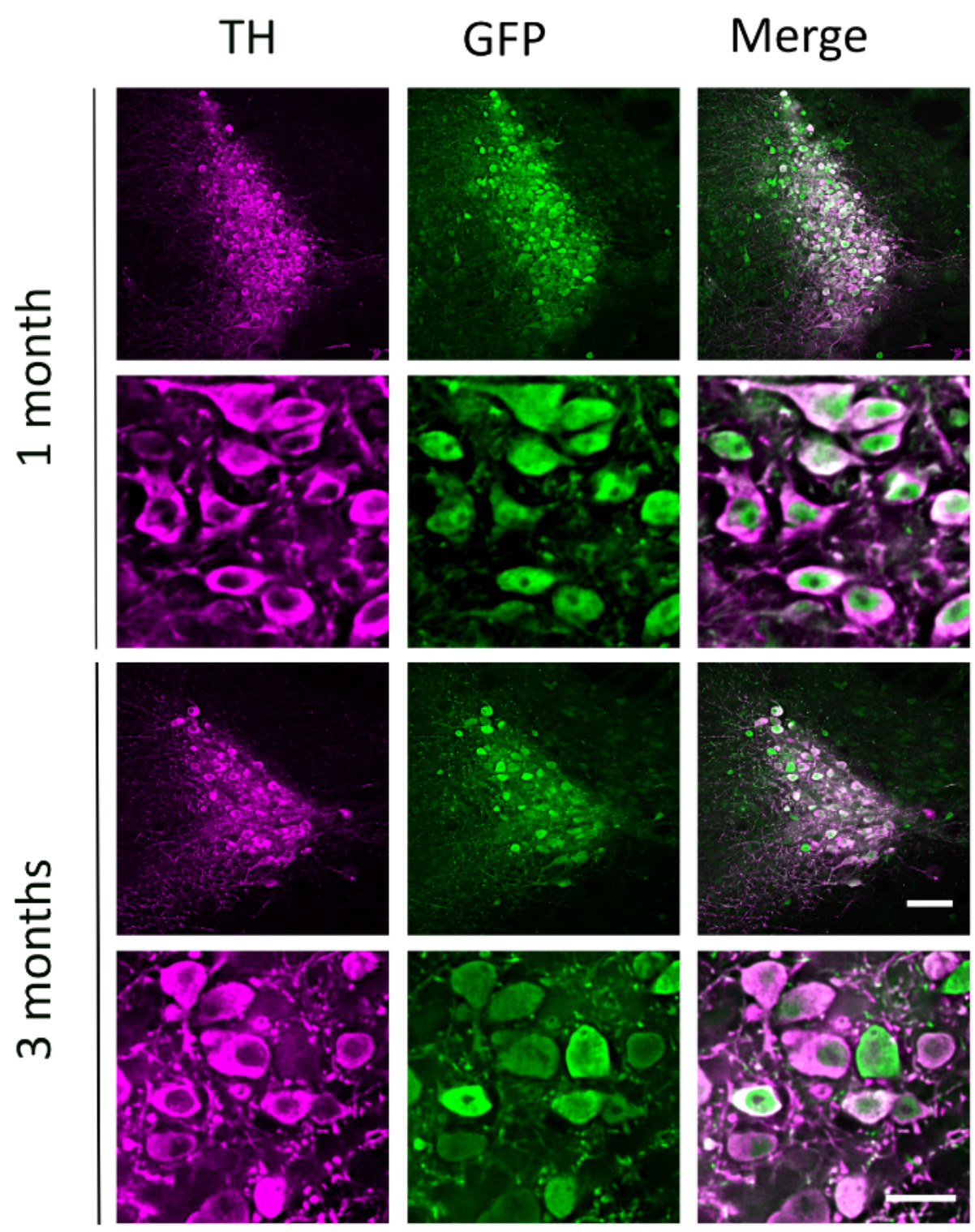

Supplementary Fig. 1. TH-GFP immunofluorescence in the Locus Coeruleus. GFP-stained neurons colocalize with TH+ neurons of the locus coeruleus. Images were captured at 20x magnification (scale bar 100 µm) and 63x magnification (scale bar 25 µm).

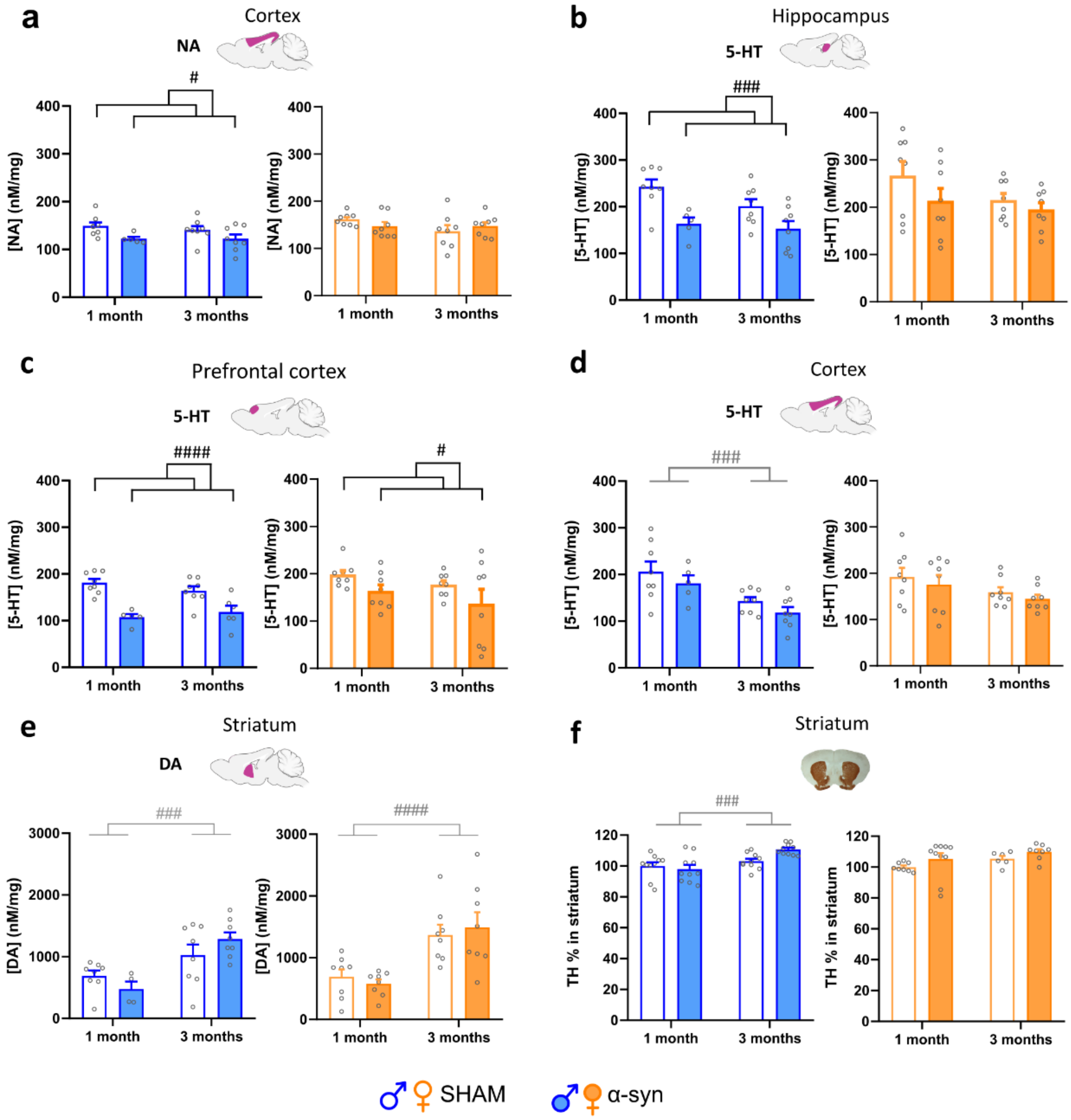

Supplementary Fig. 2. HPLC measurements of noradrenaline, serotonin and dopamine in male (blue) and female (orange) mice. (**a**) Noradrenaline levels were reduced in the cortex of male α-syn mice, with no significant changes observed in females. (**b**) In the hippocampus, serotonin levels were reduced in α-syn males but not in females. (**c**) Serotonin levels in the prefrontal cortex were decreased in both sexes in the α-syn group. (**d**) No differences in cortical serotonin levels were found between sham and α-syn mice. (**e**) No differences were found in striatal dopaminergic levels between sham and α-syn mice. (**f**) No differences were observed between the sham and α-syn groups in TH fiber expression in the striatum. N=4 animals. # $p < 0.05$, ## $p < 0.01$, ### $p < 0.001$, #### $p < 0.001$. Two-way ANOVA. Created using Biorender.

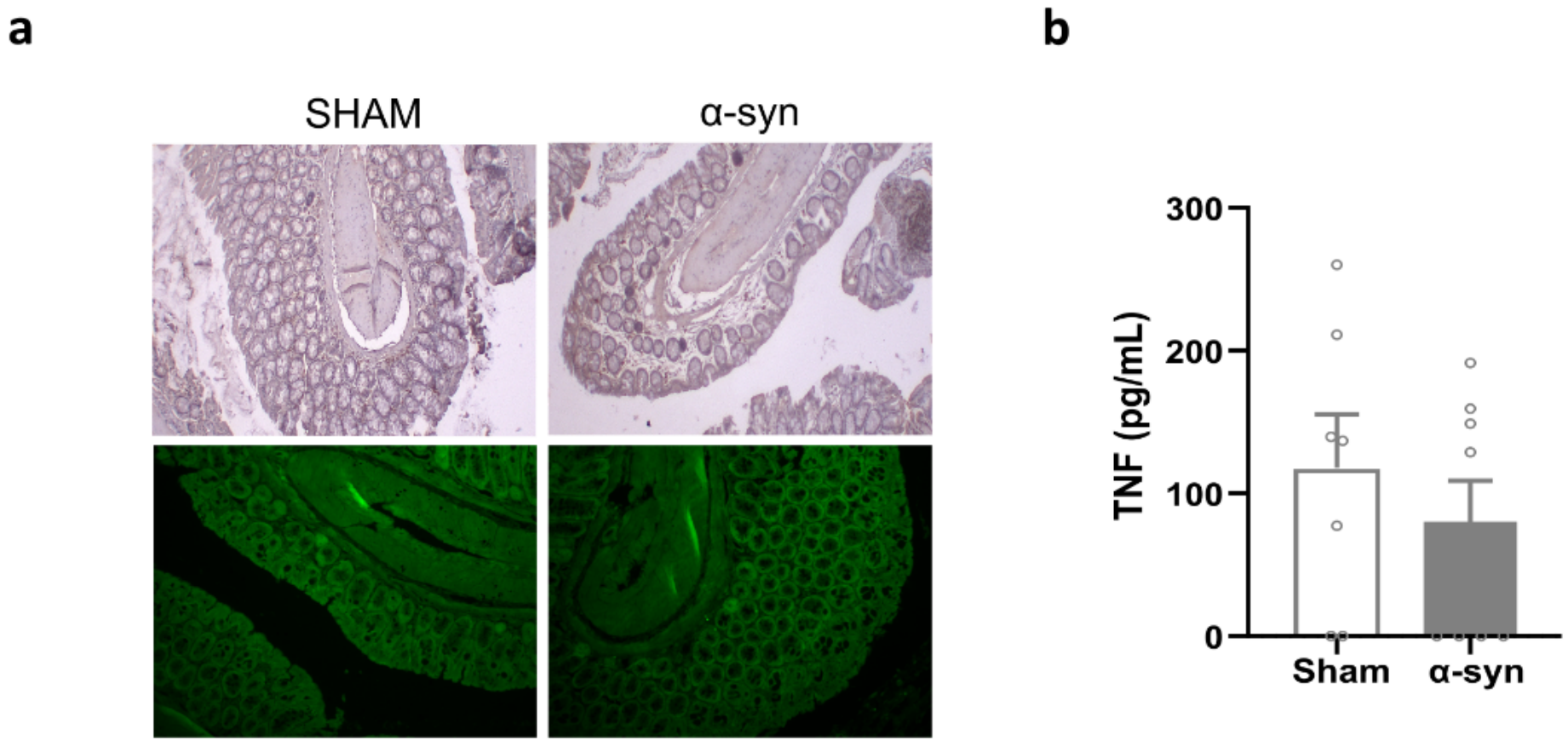

Supplementary Fig. 3. Colonic morphology and serum TNF levels in the α-syn model. (**a**) Haematoxylin staining did not reveal morphological alterations in the colons of the α-syn group (upper panel). α-syn immunofluorescence did not detect α-syn in the colon (lower panel). (**b**) Serum TNF levels showed no significant differences between the sham and α-syn groups.

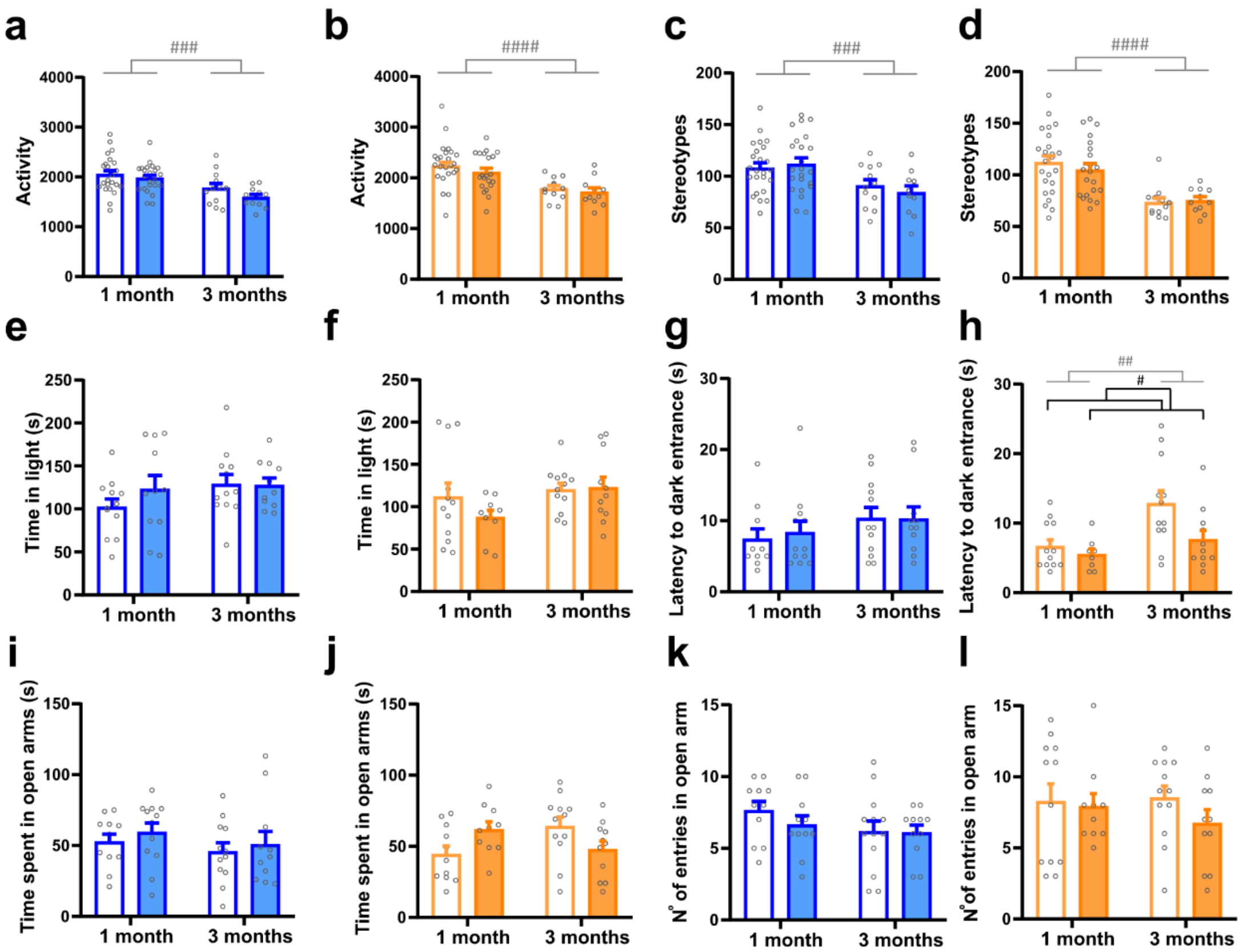

Supplementary Fig. 4. Behavioural analysis of the α-syn model in male (blue) and female (orange) mice. (**a-d**) Motor activity and stereotypy analysis in the Open Field test showed no differences among the groups. (**e-h**) Dark and Light Box test showed that α-syn mice took less time to enter to the dark compartment. (**i-l**) No differences were observed between groups in the Elevated Plus Maze test. N= 10-24 animals. # $p < 0.05$, ## $p < 0.01$, #### $p < 0.001$. Two-way ANOVA.

Table 1. Primary and secondary antibodies used in the immunoassays.

Antibody	Dilution	Reference	Purchase from
**Primary antibody**			
Rabbit anti-TH	1:2000	AB152	Sigma-Aldrich
Mouse anti-αsyn	1:10000	AB27766	Abcam
Mouse anti-αsynP	1:2000	AB51253	Abcam
Chicken anti-TH	1:2000	AB76442	Abcam
Chicken anti-GFP	1:400	AB13970	Abcam
Mouse-anti-NET	1:1500	NET05-02	MAb Technologies
**Secondary antibody**			
Goat anti-chicken Alexa fluor 555	1:400	A21437	Invitrogen
Goat anti-chicken Alexa fluor 488	1:400	AB150169	Abcam
Goat anti-rabbit Alexa fluor 488	1:400	A32731	Invitrogen
Goat anti-rabbit Alexa fluor 555	1:400	A21429	Invitrogen
Donkey anti-mouse Alexa fluor 555	1:400	A31570	Invitrogen
Streptavidine 647	1:500	S21374	Invitrogen
Biotinylated goat anti-rabbit IgG	1:200	BA100	Vector laboratories
Biotinylated goat anti-mouse IgG	1:200	BA2020	Vector laboratories

Table 2. Qualitative analysis of human α-syn expression at 1 and 3 months in a unilaterally injected model. The presence of human α-syn-positive axons was evaluated as follows: - no positive axons, + few positive axons, ++ more positive axons, +++ positive axons densely covering the region, ++++ positive cell bodies. IL: Ipsilateral side, injected side. CL: contralateral side.

Coordinate	General area	Specific area	1m IL	1m CL	3m IL	3m CL
β=+1.94 mm	*Prefrontal cortex*		++	+	++	+
β=+0.50 mm	*Striatum*		-	-	+	+
	Accumbens nucleus		+	-	+	+
	Lateral septal nucleus		+	+	++	++
	Motor cortex	*M1*	++	+	++	++
		M2	++	+	++	++
β=+0.38 mm	*Striatum*		-	-	++	+
	Lateral septal nucleus		+	+	++	++
	Bed nuclei of the stria terminalis		++	-	++	+
	Motor cortex	*M1*	++	+	+++	++
		M2	++	+	+++	++
β=-1.82 mm	*Striatum*		+	+	++	+
	Globus pallidus	*External section*	+	+	++	+
	Thalamus	*Polymodal association cortex related*	+	+	++	++
	Amygdala	*Basolateral*	+	+	++	+
		Central	+++	++	+++	++
β=-1.82 mm	*Dorsal hippocampus*	*CA1*	++	++	+++	+++
		CA2	++	++	+++	+++
		CA3	++	++	+++	+++
		DG	++	++	+++	+++
	Thalamus	*Polymodal association cortex related*	+	+	++	++
		Sensory-motor cortex related	+	+	++	++
		Subthalamic nucleus	+++	++	++	++
		Zona incerta	++	+	++	++
		Parasubthalamic nucleus	+++	+	+++	++
	Amygdala	*Basolateral*	+	+	++	++
		Central	+++	++	+++	++
β=-2.92 mm	*Ventral hippocampus*	*CA1*	+	+	+++	++
		CA2	+	+	+++	++
		CA3	+	+	+++	++
		DG	+	+	+++	++
	Substantia nigra	*Pars compacta*	++	+	++	++
		Pars reticulata	+	+	++	++
	Ventral tegmental area		++	++	++	++
β=-4.36 mm	*Superior colliculus*	*Somatosensory*	++	+	++	++
		Motor	++	+	++	++
	Dorsal raphe nucleus		+++	+++	+++	+++
β=-5.40 mm	*Cerebellum*		+	-	++	++
	Locus coeruleus		++++	+	++++	++
	Inferior colliculus		++	+	++	++
	Pons	*Pontine central gray*	+++	+	++	++
		Parabrachial nucleus	+++	++	+++	++
	Medulla		++	+	++	++

Table 3. Action potential properties of sham and α-syn male mice.

	SHAM	A-syn	n SHAM	n α-syn
**1 month**				
Threshold (mV)	-56.79 ± 2.33	-57.91 ± 2.79	23	20
AP amplitude (mV)	80.92 ± 2.38	81.25 ± 1.27	23	20
AHP amplitude (mV)	-23.45 ± 0.76	-25.46 ± 0.77	23	20
Half-width (ms)	1.04 ± 0.06	0.97 ± 0.03	23	20
**3 months**				
Threshold (mV)	-51.43 ± 3.51	-58.31 ± 2.41	16	16
AP amplitude (mV)	75.46 ± 3.39	77.44 ± 2.49	16	16
AHP amplitude (mV)	-20.06 ± 0.64	-20.03 ± 1.11	16	16
Half-width (ms)	0.95 ± 0.11	0.79 ± 0.03	16	16

Data are expressed as the mean ± S.E.M. of n cell.

Table 4. Action potential properties of sham and α-syn female mice.

	SHAM	A-syn	n SHAM	n α-syn
**1 month**				
Threshold (mV)	-50.44 ± 3.06	-61.10 ± 2.68*	26	21
AP amplitude (mV)	77.73 ± 1.85	80.99 ± 2.28	26	21
AHP amplitude (mV)	-23.52 ± 1.09	-24.16 ± 1.01	26	21
Half-width (ms)	0.91 ± 0.06	0.83 ± 0.06	26	21
**3 months**				
Threshold (mV)	-59.94 ± 2.84	-59.08 ± 2.97	19	19
AP amplitude (mV)	76.71 ± 2.06	83.73 ± 2.04	19	19
AHP amplitude (mV)	-21.94 ± 1.04	-21.25 ± 0.87	19	19
Half-width (ms)	1.39 ± 0.09	1.21 ± 0.03	19	19

Data are expressed as the mean ± S.E.M. of n cell.

*$P < 0.05$. Student’s t-test

Table 5. Statistical analysis of immunohistochemical procedures.

MALES	Interaction	Time (1 vs. 3 months)	Group (SHAM vs. α-syn)
TH+ neurons in LC	F (1, 36) = 1.88	F (1, 36) = 0.07	F (1, 36) = 0.66
TH+ neurons in SNc	F (1, 36) = 0.77	F (1, 36) = 0.00	F (1, 36) = 1.43
DBH+ area in HPC	F (1, 38) = 0.39	F (1, 38) = 13.48***	F (1, 38) = 15.78***
TH+ fibres in STR	F (1, 36) = 5.25*	F (1, 36) = 14.34***	F (1, 36) = 1.74

FEMALES	Interaction	Time (1 vs. 3 months)	Group (SHAM vs. α-syn)
TH+ neurons in LC	F (1, 36) = 3.43	F (1, 36) = 1.33	F (1, 36) = 1.12
TH+ neurons in SNc	F (1, 36) = 0.63	F (1, 36) = 0.90	F (1, 36) = 0.08
DBH+ area in HPC	F (1, 46) = 7.48**	F (1, 46) = 5.09*	F (1, 46) = 1.20
TH+ fibres in STR	F (1, 28) = 0.02	F (1, 28) = 3.35	F (1, 28) = 3.15

Two-way ANOVA-F (DFn. DFd). * p<0.05. **p<0.01, ***p<0.001.

α-syn fiber optical density IPSILATERAL	P value	P value summary	t(df)	n 1 month	n 3 months
Striatum	0.59	ns	t=0.54, df=10	6	6
Motor cortex	0.31	ns	t=1.05, df=10	6	6
Hippocampus	0.02	*	t=2.63, df=10	6	6
Substantia nigra pars compacta	0.08	ns	t=1.88, df=10	6	6
Ventral tegmental area	0.56	ns	t=0.59, df=10	6	6
Subthalamic nucleus	0.13	ns	t=1.63, df=10	6	6
Dorsal raphe	0.32	ns	t=1.02, df=10	6	6
Locus coeruleus	0.69	ns	t=0.40, df=9	6	6

Unpaired t-test. * p<0.05.

α-syn fiber optical density CONTRALATERAL	P value	P value summary	t(df)	n 1 month	n 3 months
Striatum	0.23	ns	t=1.27, df=10	6	6
Motor cortex	0.27	ns	t=1.15, df=10	6	6
Hippocampus	0.04	*	t=2.28, df=10	6	6
Substantia nigra pars compacta	0.03	*	t=2.46, df=10	6	6
Ventral tegmental area	0.60	ns	t=0.53, df=10	6	6
Subthalamic nucleus	0.36	ns	t=0.95, df=10	6	6
Dorsal raphe	0.25	ns	t=1.21, df=10	6	6
Locus coeruleus	0.30	ns	t=1.09, df=9	6	6

Unpaired t-test. * p<0.05.

Table 6. Statistical analysis of monoamine levels quantifications.

MALES		Interaction	Time (1 vs. 3 months)	Group (SHAM vs. α-syn)
NA	PFC	F (1, 23) = 0.14	F (1, 23) = 55.59****	F (1, 23) = 13.41**
	Cortex	F (1, 25) = 0.27	F (1, 25) = 0.25	F (1, 25) = 7.63*
	Hippocampus	F (1, 25) = 0.32	F (1, 25) = 9.35**	F (1, 25) = 4.32*
5-HT	PFC	F (1, 23) = 2.00	F (1, 23) = 0.08	F (1, 23) = 34.31****
	Cortex	F (1, 25) = 4.257e-005	F (1, 25) = 14.97***	F (1, 25) = 2.32
	Hippocampus	F (1, 25) = 0.92	F (1, 25) = 2.64	F (1, 25) = 15.79***
DA	Striatum	F (1, 24) = 2.921	F (1, 24) = 17.16***	F (1, 24) = 0.029

Two-way ANOVA-F (DFn. DFd). * p<0.05. **p<0.01, ***p<0.001, ****p<0.0001

FEMALES		Interaction	Time (1 vs. 3 months)	Group (SHAM vs. α-syn)
NA	PFC	F (1, 28) = 1.68	F (1, 28) = 36.12****	F (1, 28) = 0.00
	Cortex	F (1, 28) = 2.01	F (1, 28) = 1.81	F (1, 28) = 0.03
	Hippocampus	F (1, 28) = 0.58	F (1, 28) = 7.11*	F (1, 28) = 7.74**
5-HT	PFC	F (1, 28) = 0.02	F (1, 28) = 1.74	F (1, 28) = 4.20*
	Cortex	F (1, 28) = 0.00	F (1, 28) = 3.96	F (1, 28) = 0.90
	Hippocampus	F (1, 28) = 0.52	F (1, 28) = 2.25	F (1, 28) = 2.48
DA	Striatum	F (1, 28) = 0.53	F (1, 28) = 24.08	F (1, 28) = 0.001****

Two-way ANOVA-F (DFn. DFd). * p<0.05, ****p<0.0001

Table 7. Statistical analysis of peripheral changes.

MALES	Interaction	Time (1 vs. 3 months)	Group (SHAM vs. α-syn)
Colon length	F (1, 45) = 1.21	F (1, 45) = 0.23	F (1, 45) = 1.26
Spleen weight	F (1, 30) = 2.20	F (1, 30) = 4.86*	F (1, 30) = 0.04

Two-way ANOVA-F (DFn. DFd). * p<0.05.

FEMALES	Interaction	Time (1 vs. 3 months)	Group (SHAM vs. α-syn)
Colon length	F (1, 62) = 0.05	F (1, 62) = 3.42	F (1, 62) = 11.03**
Spleen weight	F (1, 32) = 0.08	F (1, 32) = 14.29***	F (1, 32) = 4.01

Two-way ANOVA-F (DFn. DFd). **p<0.01, ***p<0.001.

Table 8. Statistical analysis of locus coeruleus neurons in α-syn and sham animals at 1 month post-surgery.

MALES	P value	P value summary	t(df)	n SHAM	n α-syn
Basal firing rate (Hz)	0.85	ns	t=0.18, df=23	17	8
Half-width (ms)	0.44	ns	t=0.78, df=41	23	20
Threshold (mV)	0.75	ns	t=0.31, df=41	23	20
Peak amplitude (mV)	0.90	ns	t=0.11, df=41	23	20
Antipeak amplitude (mV)	0.07	ns	t=1.84, df=41	23	20
Membrane capacitance (pF)	0.00	**	t=2.84, df=41	23	20
Membrane resistance (MΩ)	0.01	*	t=2.45, df=41	23	20

Unpaired t-test. * p<0.05, **p<0.01.

MALES	Interaction	Factor	SHAM vs α-syn
Depolarizing currents	F (12, 492) = 6.65****	F (12, 492) = 535.3****	F (1, 41) = 4.13*
Hyperpolarizing currents	F (4, 180) = 0.40	F (4, 180) = 309.8****	F (1, 45) = 0.24

Two-way ANOVA-F (DFn. DFd). Repeated measures. ****p<0.0001.

FEMALES	P value	P value summary	t(df)	n SHAM	n α-syn
Basal firing rate (Hz)	0.03	*	t=2.28, df=24	11	15
Half-width (ms)	0.36	ns	t=0.92, df=45	26	21
Threshold (mV)	0.01	*	t=2.55, df=45	26	21
Peak amplitude (mV)	0.27	ns	t=1.12, df=45	26	21
Antipeak amplitude (mV)	0.67	ns	t=0.42, df=45	26	21
Membrane capacitance (pF)	0.00	***	t=4.23, df=45	26	21
Membrane resistance (MΩ)	0.00	***	t=3.79, df=45	26	21

Unpaired t-test. * p<0.05, **p<0.01, ***p<0.001

FEMALES	Interaction	Factor	SHAM vs α-syn
Depolarizing currents	F (12, 540) = 2.50**	F (12, 540) = 174.2****	F (1, 45) = 9.20**
Hyperpolarizing currents	F (4, 180) = 1.47	F (1.518, 68.33) = 412.1****	F (1, 45) = 0.5618

Two-way ANOVA-F (DFn. DFd). Repeated measures. * p<0.05, **p<0.01, ****p<0.0001.

Table 9. Statistical analysis of locus coeruleus neurons in male α-syn and sham animals at 3 months post-surgery.

MALES	P value	P value summary	t(df)	n SHAM	n α-syn
Basal firing rate (Hz)	0.48	ns	t=0,70, df=15	5	12
Half-width (ms)	0.21	ns	t=1.26, df=30	16	16
Threshold (mV)	0.11	ns	t=1.61, df=30	16	16
Peak amplitude (mV)	0.64	ns	t=0.47, df=30	16	16
Antipeak amplitude (mV)	0.97	ns	t=0.02, df=30	16	16
Membrane capacitance (pF)	0.19	ns	t=1.31, df=30	16	16
Membrane resistance (MΩ)	0.97	ns	t=0.04, df=30	16	16

Unpaired t-test.

MALES	Interaction	Factor	SHAM vs α-syn
Depolarizing currents	F (12, 359) = 363.5*	F (12, 359) = 363.5***	F (1, 30) = 0.19
Hyperpolarizing currents	F (4, 120) = 1.40	F (4, 120) = 328.4****	F (1, 30) = 0.38

Two-way ANOVA-F (DFn. DFd). Repeated measures. ****p<0.0001.

FEMALES	P value	P value summary	t(df)	n SHAM	n α-syn
Basal firing rate (Hz)	0.14	ns	t=1.54, df=17	7	12
Half-width (ms)	0.06	ns	t=1.90, df=36	19	19
Threshold (mV)	0.83	ns	t=0.20, df=36	19	19
Peak amplitude (mV)	0.02	*	t=2.41, df=36	19	19
Antipeak amplitude (mV)	0.61	ns	t=0.50, df=36	19	19
Membrane capacitance (pF)	0.52	ns	t=0.64, df=36	19	19
Membrane resistance (MΩ)	0.35	ns	t=0.94, df=36	19	19

Unpaired t-test.

FEMALES	Interaction	Factor	SHAM vs α-syn
Depolarizing currents	F (12, 432) = 3.39***	F (12, 432) = 382.5****	F (1, 36) = 1.14
Hyperpolarizing currents	F (4, 144) = 0.38	F (4, 144) = 321.9****	F (1, 36) = 0.02

Two-way ANOVA-F (DFn. DFd). Repeated measures. ****p<0.0001.

Table 10. Statistical analysis of behavioural tests 1 and 3 months post-surgery.

MALES		Interaction	Time (1 vs. 3 months)	Group (SHAM vs. α-syn)
Open Field	Activity	F (1, 66) = 1.49	F (1, 66) = 16.48***	F (1, 66) = 2.61
	Locomotion	F (1, 66) = 0.44	F (1, 66) = 15.70***	F (1, 66) = 2.80
	Stereotypic movements	F (1, 66) = 0.68	F (1, 66) = 12.64***	F (1, 66) = 0.05
	Distance % in central zone	F (1, 65) = 1.04	F (1, 65) = 5.35	F (1, 66) = 6.38*
EPM	Time in open arm	F (1, 43) = 0.01	F (1, 43) = 1.37	F (1, 43) = 0.70
	N of entries	F (1, 43) = 0.29	F (1, 43) = 1.88	F (1, 43) = 0.41
DLB	Time in light (s)	F (1, 43) = 0.98	F (1, 43) = 1.94	F (1, 43) = 0.80
	Latency to dark entrance (s)	F (1, 41) = 0.09	F (1, 41) = 2.38	F (1, 41) = 0.07
Sucrose preference	2h	F (1, 66) = 2.80	F (1, 66) = 0.98	F (1, 66) = 0.04
	24h	F (1, 66) = 0.30	F (1, 66) = 0.27	F (1, 66) = 0.23
Buried food task	Latency (s)	F (1, 50) = 0.20	F (1, 50) = 0.06	F (1, 50) = 0.21
Hot plate	Latency to pain behaviour	F (1, 43) = 2.81	F (1, 43) = 0.08	F (1, 43) = 2.76
NOR	Discrimination index	F (1, 63) = 1.87	F (1, 63) = 1.54	F (1, 63) = 12.21***

Two-way ANOVA-F (DFn. DFd), ***p<0.001, ****p<0.0001.

FEMALES		Interaction	Time (1 vs. 3 months)	Group (SHAM vs. α-syn)
Open Field	Activity	F (1, 64) = 0.12	F (1, 64) = 24.97****	F (1, 64) = 1.35
	Locomotion	F (1, 64) = 0.09	F (1, 64) = 22.58****	F (1, 64) = 1.37
	Stereotypic movements	F (1, 64) = 0.47	F (1, 64) = 27.29****	F (1, 64) = 0.16
	Distance % in central zone	F (1, 63) = 0.46	F (1, 63) = 8.16**	F (1, 64) = 4.77*
EPM	Time in open arm	F (1,41) = 8.174**	F (1, 41) = 0.26	F (1, 41) = 0.02
	N of entries	F (1, 41) = 0.48	F (1, 41) = 0.20	F (1, 41) = 1.08
DLB	Time in light (s)	F (1, 41) = 1.18	F (1, 41) = 3.23	F (1, 41) = 0.79
	Latency to dark entrance (s)	F (1, 39) = 2.05	F (1, 41) = 8.73**	F (1, 41) = 5.12*
Sucrose preference	2h	F (1, 64) = 0.24	F (1, 64) = 5.78*	F (1, 64) = 0.18
	24h	F (1, 64) = 0.00	F (1, 64) = 5.74*	F (1, 64) = 0.00
Buried food task	Latency (s)	F (1, 50) = 2.84	F (1, 50) = 0.08	F (1, 50) = 0.70
Hot plate	Latency to pain behaviour	F (1, 41) = 1.11	F (1, 41) = 1.30	F (1, 41) = 1.05
NOR	Discrimination index	F (1, 58) = 1.24	F (1, 58) = 0.04	F (1, 58) = 6.01*

Two-way ANOVA-F (DFn. DFd). * p<0.05, **p<0.01, ****p<0.0001.
